# Dutch guideline on total hip prosthesis

**DOI:** 10.3109/17453674.2011.623575

**Published:** 2011-11-24

**Authors:** Bart A, Swierstra, Anton MJS Vervest, Geert HIM Walenkamp, B Wim Schreurs, Pieter TJ Spierings, Ide C Heyligers, Job LC van Susante, Harmen B Ettema, Mariette J Jansen, Pim J Hennis, Janneke de Vries, Sabrina B Muller-Ploeger, Margreet A Pols

**Affiliations:** ^1^The Dutch Orthopaedic Association; ^2^the Royal Dutch Society of Physical Therapy; ^3^the Netherlands Society of Anesthesiologists; ^4^the Dutch Association for Medical Microbiology; ^5^the Department of Professional Quality, the Dutch Association of Medical Specialists, the Netherlands

Clinical practice guidelines are being used in many countries throughout the world to improve the quality of patient care. The Dutch Orthopaedic Association has a long tradition of guideline development, starting in the mid-1980s with “eminence-based consensus” and following in the mid-1990s the renewed calls for the establishment of international methodologies to promote the rigorous development of clinical guidelines and to assess their quality and their impact on practice.

This updated guideline on total hip prosthesis was developed using the “Appraisal of Guidelines for Research and Evaluation (AGREE)” instrument (www.agreecollaboration.org).

## Methods

The process started with the formulation of current questions—both from the clinician's and the patient's point of view—by a steering group whose members were the authors of this paper.

### Literature search

First, a general search was carried out for existing guidelines and systematic reviews. Afterwards, for each question the bibliographic databases PubMed and Embase were searched, using specific terms, to identify scientific literature published between 2000 and 2009. Studies published after January 1, 2009 were not included unless they would alter the conclusions. Reference lists of the retrieved studies were searched by hand for additional studies. The steering group was mainly interested in (systematic reviews of) randomized controlled trials (RCTs). If no RCTs were found, studies of a lower level of evidence were included.

### Grading of study quality

After selection of relevant literature by the steering group, the studies were graded for quality and level of evidence by a methodologist and the members of the steering group (Table 3, supplementary data). The criteria used are described in Table 1 (a systematic review of poor quality was downgraded one level). For each question, the scientific evidence was summarized in a conclusion with an accompanying level of evidence (Table 2).

**Table 1.** Grading of methodological quality of individual studies

Level of evidence	Interventional studies	Diagnostic accuracy studies	Harm, side effects, etiology, prognosis
A1	Systematic review / meta-analysis of at least 2 independently conducted studies of A2 level		
A2	Randomized, double-blind trial with good study quality and a adequate number of study participants	Indextest compared to reference test (reference standard); cut-offs were defined a priori; independent interpretation of test results; an adequate number of consecutive patients were enrolled; all patients received both tests.	Prospective cohort study of sufficient magnitude and follow-up, adequately controlled for ‘confounding’ and no selective follow-up.
B	Clinical trial, but without all the features mentioned for level A2 (including case-control study, cohort study).	Index test compared to reference test, but without all the features mentioned for level A2.	Prospective cohort study, but without all the features mentioned for level A2 or retrospective cohort study or case-control study.
C	Non-comparative studies		
D	Expert opinion		

**Table 2.** Level of evidence of the conclusion

Level	Conclusion based on
1	A1 study or at least 2 independent studies of level A2.
2	1 study of level A2 or at least 2 independent studies of level B.
3	1 study of level B or C.
4	Expert opinion.

For total hip replacement, the implant registries are an important source of information regarding outcome and factors influencing outcome. Many “population-based” registries meet the requirements of an A2 level of evidence (prospective cohort study of sufficient magnitude and follow-up, adequately controlled for “confounding” and no selective follow-up), and were graded as such.

### Recommendations

Apart from the scientific evidence, recommendations are influenced by other considerations such as patient preferences, costs, availability of facilities, or organizational aspects. The recommendations for each question are based on the scientific evidence in combination with the most important considerations.

## What are the indications and contraindications for total hip replacement?

*Scientific evidence*

Level 1:

- Younger patients and men have an increased risk of revision of their total hip prosthesis (Flugsrud et al. 2007, Santaguida et al. 2008).
- Improvement of postoperative function after total hip replacement is diminished at higher age (particularly in women) (Santaguida et al. 2008).

Level 2:

- Postoperative complications (dislocation, infection, revision) occur more frequently with obesity (Flugsrud at al. 2007, Lübbeke et al. 2007, Sadr Azodi et al. 2008).
- Men with heavy physical activities in their spare time have an increased risk of revision of the acetabular component (Flugsrud et al. 2007).

Level 3:

- Poor preoperative mobility and function do not influence postoperative pain (alleviation) (Röder et al. 2007).
- Obesity does not influence postoperative pain (alleviation), but reduces functional outcome (Busato et al. 2008).

*Consideration*. Based on demographic projection only, the number of total hip replacements in the Netherlands will increase from 20,715 in 2005 to 31,731 in 2030. Based on the continued trend, however, the number is expected to increase to 51,680 in 2005 (Otten et al. 2010). Furthermore, national and international differences in the incidence of total hip replacements due to osteoarthritis have been observed (Merx et al. 2003, Nationaal Kompas Volksgezondheid 2010). This reflects the fact that the indication for hip replacement does not only depend on the incidence and prevalence of osteoarthritis, but is also influenced by other factors such as a more active lifestyle in the elderly, higher life expectancy, improved outcomes of arthroplasties, changing reimbursement systems, etc. Thus, indications for total hip replacement differ around the world, and can only be given in general terms.

*Recommendation*. The indication for total hip replacement should be based on pain, loss of function, radiographic changes, and failure of nonoperative treatment. Younger age and obesity are relative contraindications. Delay of surgery in high age is not advisable in view of reduced functional outcome and increased mortality. In addition, when progressive loss of function (with or without contractures) predominates over pain, surgery should not be delayed in view of reduced postoperative functional outcome.

## What is the preferred type of prosthesis?

Different aspects of the total hip prosthesis are discussed separately but cannot be evaluated independently from each other in a particular prosthesis type.

### Cemented fixation vs. cementless fixation

*Scientific evidence*

Level 1:

- Several cemented and cementless femoral prostheses have a proven favorable survival (> 90% after 10–15 years), but the survival of the acetabular component is not uniform.
- Arthroplasty registers show better results for cemented prostheses than for cementless prostheses, which is mainly due to inferior results of some cementless acetabular components (National Joint Registry UK 2007, Australian Orthopaedic Association 2008, Mäkelä et al. 2008, Norwegian Arthroplasty Register 2008).

Level 3:

- Expensive prostheses need much better results to achieve economically cost-effective benefits, especially in patients aged 50–70 years (Fitzpatrick 1998).

*Considersation*. The results for cementless prostheses are mostly based on studies in young patients. Comparable results are obtained if the factor age is adjusted for, although studies on cementless prostheses reveal more revisions for change of the polyethylene liner (Mäkelä et al 2008). The culture of developing and marketing new hip prostheses reflects a high level of innovation and experimentation, but also commercial interests. An economically based study concluded that a new prosthesis costs about 3 times more than a standard prosthesis and is only cost-effective if the revision rate is reduced by about 40% (Fitzpatrick et al. 1998).

*Recommendation*. The choice of a total hip prosthesis, cemented or uncemented, must be based on peer-reviewed published studies with a follow-up of at least 10 years, and on the (direct and indirect) costs. New implants should be introduced according to 4 steps: laboratory studies, small clinical series using radiostereometry, randomized studies compared with a well-documented prosthesis, and finally follow-up in an implant registry.

### Head diameter

*Scientific evidence*

Level 1:

- In traditional metal-on-polyethylene articulations, 32-mm heads show higher wear rates than 22- or 28-mm heads after 10 years of clinical use (Oparaugo et al. 2001, Tarasevicius et al. 2006).

Level 2:

- The incidence of posterior dislocations is lower in 32-mm heads than in 22- or 28-mm heads (Bystroem et al. 2003).
- Short-term clinical outcome data (up to 5 years) for head diameters greater than 32 mm are comparable to the clinical outcome data for 22-, 28-, or 32-mm heads, but with lower dislocation rates within the first 3 months after surgery (Amstutz et al. 2004, Cuckler et al. 2004, Smith et al. 2005, Geller et al. 2006, Peters et al. 2007, Sikes et al. 2008).

*Consideration*. The reason for dislocation of a total hip prosthesis is multifactorial and related to the patient, the surgeon, the surgical approach, the type of prosthesis, and the head size. In traditional metal-on-polyethylene bearings, 32-mm heads have lower dislocation rates; however, the lowest wear rates are seen in 22-mm heads. To prevent dislocation, there is a trend toward larger head diameters, which is supported by alternative bearings (metal or ceramic on crosslinked polyethylene, metal-on-metal, ceramic-on-ceramic) that are more wear-resistant. The outcome data up to 5 years for larger heads are comparable to those for head diameters of 32 mm or less, but long-term data are needed. Recently, there have been some concerns about the claimed wear resistance of the crosslinked polyethylenes in combination with with larger heads (Lachiewicz et al. 2009). Also, the theoretical advantage of larger heads is limited in practice because surgeons have the tendency to place the larger cups too vertically (Crowninshield et al. 2004).

*Recommendation. *More clinical and long-term evidence is needed to justify the standard use of larger-diameter heads. Heads larger than 32 mm should be restricted to patients with a high risk of dislocation. Other indications are preferably used in a clinical study setting.

### Bearing

*Scientific evidence*

Level 1:

- The application of crosslinked polyethylene reduces the wear of polyethylene acetabular cups and inserts in the medium term. There is as yet no evidence that crosslinking improves the survival rate of total hip prostheses (Triclot et al. 2007, Garcia-Rey et al. 2008, Geerdink et al. 2009, McCalden et al. 2009, Rajadhyaksha et al. 2009).
- The reduced wear of ceramic-on-polyethylene bearings in comparison to metal-on-polyethylene bearings is not reflected by improved clinical results in the medium term. (Kim 2005, Kraay et al. 2006).
- Metal-on-metal bearings cause increased serum levels of metal ions (Brodner et al. 2003, Dahlstrand et al. 2009).
- The reduced wear of ceramic-on-ceramic bearings in comparison to other common bearings does not result in improved clinical results in the long term. (Bierbaum et al. 2002, D'Antonio et al. 2005, Seyler et al. 2006, Capello et al. 2008, Lewis et al. 2010).

*Consideration.* The efficacy of various combinations of soft and hard bearing materials is commonly measured in terms of wear rate. Polyethylene acetabular components show less wear if small head diameters are used. Wear of polyethylene can also be reduced by the use of crosslinked polyethylene. Hard material combinations such as metal-on-metal or ceramic-on-ceramic rely on hydrodynamic lubrication. Their wear rate is less than that of polyethylene bearings, even if large-diameter heads are used. One benefit of large head diameter is a reduced dislocation rate. The performance of hard bearings is dependent on component positioning. There is little evidence for any clinical benefit of using hard bearing materials. Metal-on-metal bearings consistently show elevated serum levels of metal ions. In the Australian Orthopaedic Association National Joint Replacement Registry (2008), metal-on-polyethylene bearings have had a lower revision rate than all other combinations of bearing materials (after correction for age and sex).

*Recommendation.* A metal or ceramic head and a conventional polyethylene acetabular cup or liner would be the first choice. Based on the medium-term reduced wear, a crosslinked polyethylene cup or liner can be considered. There is insufficient evidence to support the use of other types of bearings, and we recommend that they should be used for investigational purposes only.

## What is the value of resurfacing hip arthroplasty?

*Scientific evidence*

Level 2:

- The short-term functional outcome of resurfacing hip arthroplasty is comparable to that after a conventional total hip arthroplasty (Pollard et al. 2006, Marker et al. 2009, Mont et al. 2009, Fowble et al. 2009, Lavigne et al. 2010, Stulberg et al. 2010).
- Resurfacing hip arthroplasty has some advantages over a conventional total hip replacement: a relatively higher activity score can be established and dislocations are rather uncommon. There is (still) no evidence that the bone-preserving nature of the procedure is of clinically relevant benefit in revisions (Pollard et al. 2006, Vail et al. 2006, Fowble et al. 2009, Mont et al. 2009, Lavigne et al. 2010).
- Resurfacing hip arthroplasty has clinically relevant disadvantages over conventional total hip replacement. Without proper patient selection, the early revision rates are higher than after a conventional total hip arthroplasty. The most frequent causes of revision are aseptic loosening, femoral neck fracture, and adverse reactions to metal-on-metal particle release (Glyn-Jones et al. 2009, Grammatopolous et al. 2009, Kahn et al. 2009, Prosser et al. 2010).

*Consideration. *A good result with hip resurfacing depends on a combination of adequate patient selection, experience with the relatively complex surgical technique, and choice of implant. In the last few years, there has been increasing concern about toxic effects of focal and systemic metal ion exposure from these implants. A global decrease in the number of implanted resurfacing hip arthroplasties can be noted in the national registries. Only with a thoroughly performed long-term follow-up—preferably in national implant registries—will the true advantages and disadvantages of hip resurfacing in the young patient with osteoarthritis of the hip be elucidated.

*Recommendation.* Resurfacing hip arthroplasty should be performed under close monitoring of the results and should be reserved for relatively young patients (below 60–65 years of age) with a femoral head diameter of greater than 50 mm and good bone stock. Data from national implant registries should dictate the choice of implant and the surgeon should have good experience of the relative complex surgical technique.

## What is the preferred surgical approach for total hip replacement?

### Conventional procedures

*Scientific evidence*

Level 2:

- There is no difference in postoperative function between the posterolateral, the straight lateral, the anterolateral, and the anterior approaches to the hip (Masonis et al. 2002, Jolles et al. 2006, Kwon et al. 2006).
- The straight lateral approach gives the lowest dislocation rate (Masonis et al. 2002, Jolles et al. 2006, Kwon et al. 2006).
- Repair of the capsule diminishes the dislocation rate of the posterolateral approach (Masonis et al. 2002, Kwon et al. 2006).

*Recommendation.* There is no preference for any of the 4 surgical approaches. Repair of the capsule is advised in the posterolateral approach.

### Minimally invasive procedures

*Scientific evidence*

Level 1:

- Minimally invasive total hip replacement has short-term advantages such as faster recovery and therefore shorter hospital stay (Mahmood et al. 2007, Verteuil et al. 2008, Wall et al. 2008, Chen et al. 2009).

Level 2:

- Minimally invasive hip surgery causes more muscle damage and a cosmetically inferior (though smaller) scar (Mardones et al. 2005, Mow et al. 2005, Goldstein et al. 2008).

Level 3:

- The advantages of minimally invasive hip surgery are mainly due to quicker rehabilitation and better postoperative pain control (Nuelle et al. 2007).

*Consideration.* Many total hip prostheses with proven good long-term results are not suitable for minimally invasive hip surgery (MIS), so there is a tendency to use implants without proven durability. The popularity of MIS is based on short-term advances such as shorter recovery time. Nuelle et al. (2007) concluded that patients operated by the traditional approach who had fast rehabitation programs recovered as quickly as patients treated by MIS.

*Recommendation.* Minimally invasive hip surgery should be restricted to controlled studies, as it is not yet clear whether the short-term advantages balance the possible long-term disadvantages.

## What is the preferred method to prevent postoperative thromboembolic complications?

*Scientific evidence*

Level 1:

- The incidence of thromboembolic complications following total hip arthroplasty can be adequately reduced with low molecular weight heparins, fondaparinux, dabigatran, vitamin K antagonists, and rivaroxaban.
- Extended out-of-hospital thromboprophylaxis can further reduce the rate of venous thromboembolism following hip arthroplasty (Eriksson et al. 2007, 2008, Geerts et al. 2008, Kakkar et al. 2008).

*Consideration.* Several methods (mechanical and pharmacological) to reduce the incidence of venous thromboembolism (VTE) are available. Mechanical methods are generally less effective than pharmacological thromboprophylaxis, and are cumbersome when used out of hospital. Thus, the use of pharmacological prophylaxis is advised except when a high risk of bleeding precludes the use of pharmacological agents. There appears to be no difference between a preoperative and a postoperative start of thromboprophylaxis regarding efficacy and bleeding risk. A preoperative start is probably more effective, but is counterbalanced by an increased bleeding risk (Strebel et al. 2002). The risk on VTE continues to increase for a prolonged period, even after hospital discharge (White et al. 1998).

*Recommendation. *Low molecular weight heparins, fondaparinux, dabigatran, vitamin K antagonists, or rivaroxaban are effective means to prevent thrombosis after total hip replacement. Thromboprophylaxis can be initiated postoperatively and continued for 4–5 weeks after surgery. Adequate monitoring of side effects is advised when new anticoagulants (dabigatran, rivaroxaban) are used.

## What prophylactic measures against infection should be used in primary total hip replacement?

### Systemic antibiotics

### Scientific evidence

### Level 1:

- Systemic antibiotics are effective in the prevention of deep and superficial infection, with a relative risk reduction of about 80% (AlBuhairan et al. 2008, Gillespie and Walenkamp 2010).
- There is no difference in efficacy between first- and second-generation cephalosporines (Albuhairan et al. 2008).
- Antibiotics must be given 15–60 min before the incision (Classen et al. 1992, Bowers et al. 1973, Kasteren et al. 2007, Stefansdóttir et al. 2009, Steinberg et al. 2009).
- The maximum duration of antiobiotic prophylaxis is 24 h (AlBuhairan et al. 2008, Gillespie and Walenkamp 2010).

Level 4:

- In cases of high risk of MRSA (as in carriers), a glycopeptide (teicoplanin or vancomycin) should be used (Soriano et al. 2006, Meehan et al. 2009).

### Antibiotic-loaded bone cement

*Scientific evidence*

Level 2:

- In cemented prostheses, the use of antibiotic-loaded bone cement has a prophylactic effect on deep infections. The rate of “aseptic” loosening is also reduced, possibly by reduction of low-grade infections (Josefsson et al. 1993, Espehaug et al. 1997, Malchau et al. 1998, Parvizi et al. 2008).
- The incidence of superficial wound infections is not reduced by antibiotic-loaded bone cement; prophylaxis with systemic antibiotics remains necessary (Josefsson et al. 1993).
- Prophylactic administration of systemic antibiotics and antibiotic-loaded bone cement reduce the risk independently and can be combined (multiplied) (Espehaug et al. 1997, Persson et al. 1999, Engesaeter et al 2003).

### Air-handling systems

*Scientific evidence*

Level 1:

- When prostheses are implanted, the air supplied at the operating area and the instrument tables must contain less than 10 cfu bacteria per m3 (Lidwell et al. 1982, Malchau et al. 1993).

*Consideration.* Antibiotics are the most effective prophylactic measure for prevention of infection. The risk reduction is 75–80%. They must be active against the most frequent causative bacteria: S. aureus and S. epidermidis. The maximum duration of the prophylaxis is 24 h. Whether or not 1 dose is sufficient is debated. In prosthesis implantation, a duration of 12–24 h seems better, also since postoperative pneumonia and urinary tract infection are reduced, as well as aseptic loosening (Wymenga et al. 1992, Engesaeter et al. 2003, Gillespie and Walenkamp 2010). When antibiotics are administered too late, the tissue concentration will be too low; given too early, the antibiotic concentration will be too low at the end of the operation—especially for antibiotics with a short half-life.

Antibiotic-loaded bone cement has a protective effect by release of the antibiotic from the surface. In animal experiments, this prophylaxis is effective 6 weeks postoperatively (Elson et al. 1977, Blomgren 1981), so it protects against both peroperative contamination and early postoperative bacteremia that may cause hematogenous infection. In general, the commercially available bone cements—often using gentamicin—are effective.

Prevention of contamination of the wound is the most effective and logical measure. There is a direct relationship between the amount of bacteria in the air and the deep infection rate (Lidwell et al. 1982). In prevention of contamination, other measures such as occlusive clothing and strict discipline regarding hygiene are equally important, but they cannot compete with the effect of uncontaminated air. Clean air reduces bacterial contamination of the wound, and has proven to be highly effective. The best choice is a laminar downflow displacement ventilation system with a large plenum (3 × 3 m^2^), an air inflow speed of 35 cm/sec, and with the inlet air 2 degrees colder than the outlet air.

General prophylactic measures are assumed to be applied—such as disinfection, occlusive clothing, strict discipline, and optimal surgical technique (Knobben et al. 2006). Systemic antibiotics, local antibiotics, and clean air reduce the risk of infection by 80%, 50%, and 50% respectively, and they act independently of each other (Lidwell et al. 1987). These means of reduction of infection risk can be combined, however (Persson et al. 1999).

*Recommendation.* In all primary total hip replacements, systemic antibiotic prophylaxis is advisable, with first- or second-generation cephalosporines started 15–60 min before incision and continued for 24 h at most, and when cemented in combination with the use of antibiotic-loaded bone cement. Furthermore, the operating room should be supplied with a modern displacement ventilation system that is capable of maintaining bacterial counts of less than 10 cfu/m^3 ^in the operation field.

## How does one prevent hematogenous infection of prostheses?

*Scientific evidence*

Level 2:

- Hematogenic infections of prostheses occur mainly in patients with reduced immunity, especially rheumatoid arthritis, and particularly in cases of skin infection in the same leg (Deacon et al. 1996, Kaandorp 1998, Krijnen et al. 2001).
- Patients with an active infection in their body have a higher risk of prosthetic infection (Ainscow et al. 1984, Deacon et al, 1996, Waldman et al 1997, Kaandorp 1998, Krijnen et al. 2001).

Level 3:

- Antibiotic prophylaxis in dental procedures is only useful when these procedures are performed on infected tissue (Gillespie 1990, Krijnen et al. 2001).

*Consideration.* Bacteremia is common, but may only cause hematogenous infection of the prosthesis when the bacterial load is high and the bacteria are virulent. The most frequent causes are skin infections (Deacon et al. 1996, Kaandorp 1998). Advisory committees in several countries came to the same conclusion: only give antibiotic prophylaxis in dental treatment when performed in an infected region (Uçkay et al. 2008).

*Recommendation.* Prophylactic antibiotics (e.g. 1,250 mg amoxicilline/clavulanic acid) should be given in all invasive procedures in patients with reduced immunity, in dental procedures in infected tissue, in endoscopy and cystoscopy in symptomatic infections, and in esophagoscopy.

## What is the preferred anesthetic technique for total hip replacement?

*Scientific evidence*

Level 1:

- Less postoperative pain is experienced after regional anesthesia than after general anesthesia (MacFarlane et al. 2009, Choi et al. 2009).
- Blood loss and the incidence of venous thrombosis are not different in regional and general anesthesia (Mauermann et al. 2006, MacFarlane et al. 2009, Choi et al. 2009, Hu et al. 2009).

Level 1–2:

- Neuraxial anesthesia (spinal or epidural) results in urinary retention and hypotension more often than does general anesthesia (Choi et al. 2009).

*Consideration.* Regional techniques consist of neuraxial analgesia or peripheral nerve blockade. The duration of surgery, length of hospital stay, cardiopulmonary morbidity, incidence of thromboembolic events, cognition and blood loss were no different in either of the techniques used compared with general anesthesia. Pain, nausea, and vomiting were reduced in patients who had undergone regional techniques.

*Recommendation.* Regional anesthetic techniques are to be preferred, based on better quality of postoperative analgesia. When neuraxial anesthesia is used, urinary retention is a risk; this can be effectively reduced through the use of a urinary catheter after surgery.

## What is the value of physiotherapy?

*Scientific evidence*

Level 2:

- Physiotherapy after total hip replacement is effective for recovery of strength, physical function, and stability (Suetta et al. 2004, Trudelle et al. 2004, Maire et al. 2006, Galea et al. 2008).
- Physiotherapy before total hip replacement is not effective for recovery of physical function and reduction of pain (Gocen et al. 2004, Rooks et al. 2006, Ferrara et al. 2008).
- Clinical pathways in total hip replacement are cost-effective, while functional outcomes and complications are comparable (Kim et al. 2003, Brunenberg et al. 2005, Siggeirsdottir et al. 2005, Larsen et al. 2008).

*Consideration.* Generally speaking, preoperative exercise is not effective, but because poor function is a risk factor for poor recovery after total hip replacement, preoperative training may be considered in (older) dependent patients with poor function. During hospital stay, postoperative rehabilitation after total hip replacement is aimed at quick mobilization guided by local hospital protocols. After discharge from the hospital, postoperative physiotherapy is continued with the purpose of counteracting physical dysfunction, reduced strength, and reduced mobility, and to reach the patient's optimal function. There have been a few random controlled trials that studied the effects of postoperative exercise programs after total hip replacement. All the trials compared different supervised (home) exercise programs and found that they had effects on strength and physical function. So, postoperative physiotherapy is indicated in patients with total hip replacement in order to follow a supervised (home) exercise program that is based on the patient's dysfunctions. Clinical pathways are cost-effective, with comparable clinical outcomes and complications, but it is not clear whether group-oriented rehabilitation is better.

*Recommendation.* Preoperative physiotherapy (including advice and support in cane walking) may be considered only in older, dependent people with poor physical function. Postoperative physiotherapy is recommended, including a post-discharge supervised (home) exercise program that is based on the patient's dysfunctions in strength, physical function, and mobility. Complete care in total hip replacement is preferably given as clinical pathway with preoperative education about fast track aspects, individual advice and support, and postoperative rehabilitation.

## Is there a need for routine follow-up after total hip replacement?

*Scientific evidence*

Level 3:

- There is no need for routine follow-up between 1 and 5 years after total hip replacement (Röder et al. 2003, King et al. 2004).

*Consideration. *Monitoring of patients shortly after the operation concentrates on healing of the wound and on recovery of function. Broadly speaking, this stage is complete 1 year after surgery, including the fixation of an uncemented prosthesis. After the first year, routine follow-up is directed at detection of complications such as polyethylene wear or osteolysis, and deterioration of function. By being followed up routinely every 1, 2, or 3 years, patients get used to regular follow-up at a later stage. Furthermore, it can be important for an (inexperienced) orthopedic surgeon to know the results of his/her own work (quality control). This is only possible by regular clinical and radiological monitoring of his or her own patients.

*Recommendation.* Routine follow-up should be carried out at least during the first year and after the fifth year, or earlier if the surgeon considers it necessary—based on experience of the prosthesis used.

## Supplementary data

Table 3 is available at our website (www.actaorthop.org), identification number 4746.
