# Supplementary material for: Dutch guideline on total hip prosthesis
Source: Acta Orthop. 2011 Nov 24;82(5):567–76. doi: 10.3109/17453674.2011.623575 (PMC3242953; doi:10.3109/17453674.2011.623575)
Supplement: Supplementary file 1 [file ORT-1745-3674-82-567-s4746.pdf]

Supplementary article data

Dutch guideline on total hip prosthesis

Bart A Swierstra[1], Anton M J S Vervest[1], Geert HI M Walenkamp[1], B Wim Schreurs[1], Pieter T J Spierings[1], Ide C Heyligers[1], Job L C van Susante[1], Harmen B Ettema[1], Mariette J Jansen[2], Pim J Hennis[3], Janneke de Vries[4], Sabrina B Muller-Ploeger[5], Margreet A Pols[5]

[1]The Dutch Orthopaedic Association; [2]the Royal Dutch Society of Physical Therapy; [3]the Netherlands Society of Anesthesiologists; [4]the! Dutch Association for Medical Microbiology; [5]the Department of Professional Quality, the Dutch Association of Medical Specialists, the Netherlands
Correspondence: b.swierstra@maartenskliniek.nl
Submitted 11-03-15. Accepted 11-04-25

Open Access - This article is distributed under the terms of the Creative Commons Attribution Noncommercial License which permits any noncommercial use, distribution, and reproduction in any medium, provided the source is credited.
DOI 10.3109/17453674.2011.623575

Table 3. Scientific evidence

Which are the indications and contra-indications for total hip replacement?

Reference	Level of evidence	Study type	Number of patients	Patient characteristics	Inclusion criteria	Intervention	Control	Follow-up	Outcome measures	Results	Remarks
Santaguida et al., 2008	A2	SR	64 studies (total) 555 - 96675 patients (THR)	-23 studies ◊ knee replacement<br>-38 studies ◊THR<br>-3 studies◊ beide<br><br>Years of publication: 1989 -2002; 60% published between 1997 and 2001.<br><br>Studies based on: registries (*n*=19), administrative (e.g. Medicare) or state discharge databases (*n*=4) or large urban hospitals (*n*=4).<br><br>6 of 64 studies were prospective (including 1 multicentre randomized trial); other studies were retrospective.	-studies with sample sizes of 500 or more.<br>-of the 441 non-English studies 73% were French (26.1%), German (22.5%), Scandinavian (14.1%) or Italian (10.0%).<br><br>At least 1 of the following outcome measures: function and health, pain, revision, postoperative complications, mortality.<br><br>Prognostic factors: age, sex, race, height, weight (or BMI), SES, work status, high physical demands.<br><br>Maximum of 30% lost for mean follow-up time of 10 yr<br><br>*Inclusions*<br>• Humans with knee or hip joint arthroplasty	-	-	Range of 6 weeks to 20 years postoperative (THR)	Revision<br><br>Mortality<br><br>Function<br><br>Pain	Revision (all causes) was reported in 28 studies. Of these, 14 reported hip data stratified by age, sex and obesity. ◊ age and sex were the most consistently evaluated prognostic factors. In paticular, younger men had a higher risk of revision.<br><br>Of 16 studies in which mortality was evaluated ◊5 extractable data. Mortality intervals reported for THR were 30 days, 60 days, 90 days and 1 year.<br>Older age at time of surgery was associated with increased postoperative mortality. Male sex was also associated with higher mortality rates in all but 1 study.	Raters were not blinded to citation identifiers (e.g., author, institution, year of publication). It is not clear how many RCTs were included in the systematic review. No meta-analysis.

Reference	Level of evidence	Study type	Number of patients	Patient characteristics	Inclusion criteria	Intervention	Control	Follow-up	Outcome measures	Results	Remarks
					**arthroplasty<br>• 90% of study participants with osteoarthritis diagnosis<br>OR<br>• The% of osteoarthritis patients is less than 90% but there was a minimum of 500 patients and the subsequent analyses were stratified with respect to this diagnosis**				**Satisfaction**	**Of 17 studies that reported function ◊ 7 extractable data.<br>Older patients and women had poorer functie and less improvement relative to baseline function.<br><br>Pain was was seldom reported separately.<br>*Visuri et al.:* women experienced less postoperative pain than men.<br><br>*Stickles et al:* satisfaction did not differ for obese<br>Patients after THR.<br>*Esephauget al:* age and sex did not affect the satisfaction level of<br>patients undergoing primary hip arthroplasty.<br>However, women and older patients undergoing revision were reported to be less satisfied**	
**Lubbeke et al., 2007**	**A2**	hospital-based prospective	**2186 (2 495**	**<u>Women (n=1217)</u>**	**Primary THR**	**Incidence of main**	**Incidence of main**	**Follow-up period**	**Main complication (infection, luxation**	**<u>Obese vs non-obese</u>**	**Obese patients were**

Reference	Level of evidence	Study type	Number of patients	Patient characteristics	Inclusion criteria	Intervention	Control	Follow-up	Outcome measures	Results	Remarks
**al., 2007**		cohort study	**(2.495 hips) 589 THR in 508 obese patients. Obese men: 26.7% Obese women: 20.5%**	***BMI< 30***<br>N= 1095 (57,5%)<br>***BMI ≥ 30***<br>N= 287 (48,7%)<br><u>Men (n= 969)</u><br>***BMI< 30***<br>N= 811 (42,5%)<br>***BMI ≥ 30 (n=52)***<br>N= 302 (51,3%)<br><u>**Age at operation: Mean ± SD years**</u><br>***BMI< 30***<br>69,0 ± 12,5<br>***BMI ≥ 30***<br>67,2 ± 9,9	**between March 26, 1996 and July 31, 2005**	**main complication (infection, luxation, revision) in obese individuals.**<br><br>**Also function and patient satisfaction 5 years postoperative, stratified for sex.**	**main complication (infection, luxation, revision) in non-obese individuals.**	**period through October 31, 2005**<br><br>**5 years postoperative (n=817)**	**(infection, luxation, revision)**<br><br>**Function (Harris Hip Score and Western Ontario and McMaster Universities Osteoarthritis Index)**	<u>**obese**</u><br>**- Infection: adjusted incidence rate ratio ◊** 4,4 95% CI 1,8-10,8.<br>**Women:**<br>**- incidence rate ratio for infection comparing obese with nonobese women ◊ 16.1 (95% CI 3.4 - 75.7).**<br>**Men:**<br>**- incidence rate ratio for infection comparing obese with nonobese men ◊ 1,0; 95% CI 0,2-5.3.**<br><br>**-Dislocation: adjusted incidence rate ratio ◊**<br>2,4 95% CI 1,4-4,2<br><br>**Outcomes of function and satisfaction were moderately lower in obese women than in nonobese women, partly because of higher complication rates. Men: less difference in function and no difference in satisfaction between obese and nonobese.**	**were younger with slightly lower preoperative functional status (differences were greater in women) and higher ASA scores.**<br><br>**A2 rating because of sufficient number of patients, adequate control of confounding and no selective follow-up.**

Reference	Level of evidence	Study type	Number of patients	Patient characteristics	Inclusion criteria	Intervention	Control	Follow-up	Outcome measures	Results	Remarks
**Sadr Azodi et al., 2008**	**B**	Observational (data from Swedish registration and Swedish Construction Workers' cohort)	**2,106 patients**	**BMI categories**<br>**18,5- 24,9 n= 681**<br>**25–29,9 n= 1132**<br>**≥ 30 n= 282**	**Men with primary THR between 1997 and 2004.**	**Overweight, obesity and tobacco use.**	**No overweight, obesity and tobacco use.**	**Max. 8 years**	**The relation between BMI and tobacco use and implant luxation in THR.**	**Implant luxation:**<br>**53 patients (2,5%) developed dislocation during a mean of 2 (0–3) years of follow-up.**<br><br>**Overweight and obesity were associated with higher risk:**<br>**HR = 2,5 95% CI: 1.1–5.5 (overweight) and HR =** 3,7 95% CI: 1.5–9,3 (obesity) **compared to normal weight.**<br><br>**No significant association was found between tobacco use and risk of implant luxation.**	**No blinding of outcome assessor**<br><br>**Not clear whether groups were comparable.**
**Flugsrud et al, 2007**	**B**	Cohort	1535	**Women n= 969**<br>**Men n= 566**<br>**Mean age at screening: 49 years**<br>**Mean age at primary THR: 63 years**<br>**Mean age at end of follow-up: 69 years**	**-Norwegian Arthroplasty Register**<br><br>**-Cox regression analysis was used to estimate relative risks (RRs).**	**Risk factors overweight and high level of physical activity. Combined with age and sex ◊ risk of revision**	**-**	**Follow-up was time between primary THR and event or censoring.**	**Relative risk (RR) (Event was defined as implant revision due to aseptic loosening of cup, stem or both.)**	**Men were at greater risk than women of loosening of the femoral stem** (RR 2.0, 95% CI 1.3–3.2).<br>**Both men and women with upper-quartile body weight were at increased risk of revision due to loosening of the stem (RR 2.5 and 2.7, respectively).**<br>**Men with a high level of physical activity during leisure time were at increased risk**	**No blinding of outcome assessor and randomization.**

Reference	Level of evidence	Study type	Number of patients	Patient characteristics	Inclusion criteria	Intervention	Control	Follow-up	Outcome measures	Results	Remarks
										of revision due to loosening of the cup (RR 4.8, 95% CI 1.3–18). In the multivariate model with adjustment for activity, there was little association between age at primary THA and risk of revision due to loosening.	
**Busato et al., 2008**	**B**	Cohort study	18,968 patients<br><br>20,553 hips	**THR between 1965 and 2003 in 42 European and 1 Canadian hospital. Bilateral ◊ 7,7% of de THR _ n=10,138 (53,5%) _ n=8,830 (46,5%) Mean age at THR: 64,8 (95%CI: 65,7-65,0) years Mean BMI at THR: 26,49 (95%CI: 26,42-26,55); median: 26,2 kg/m^2**	**<u>Exclusion</u><br>- radiographic signs of loosening of prosthetic components<br>-revisions**	**Different groups based on BMI<br><br>Underweight: BMI <18,5,<br>Normal: BMI 18,5 - <25,0<br>Overweight: BMI 25,0 - <30,0<br>Obese I: BMI 30 - <35,0<br>Obese II: BMI 35,0 - <40,0<br>Extremely obese: BMI ≥ 40**	**Different groups based on BMI**	**15 years (formal statistical tests at 3, 6, 9, 12 years after THR)**	**Pain<br><br><br><br>Function**	**No significant difference between BMI groups for postoperative pain during entire follow-up. Significant difference in function between obese and normal weight (12 yrs follow-up; P< 0,05)**	**BMI significantly lower in women compared to men (women: 26,04 (95%CI: 25,94-26,14) kg/m^2; men 26,99 (95%CI: 26,91-27,08) kg/m^2) Results are adjusted for sex and diagnosis.<br><br>Too little information to exclude selection bias (completely).<br><br>No selective loss-to-follw-up.**
**Roder et al., 2007**	**B**	retrospective cohort study	**12925 patients<br><br>13766 hips**	**International Documentation and Evaluation System European hip registry, between 1967en 2002 (65 hospitals in 8 europese landen)**	**-primary total hip arthroplasty<br>-one or more complete follow-up examinations<br>-with three inclusion criteria:<br>1)age >20 years<br>2)diagnosis of**	**-**	**-**	**Max. 10 years<br><br>Mean number of follow-up visits per patiënt:<br>2,1 (range, 1-9)<br><br>Mean duration of follow-up:**	**<u>Pain</u> was classified as none/mild, moderate, or severe/intolerable; <u>walking</u> capacity was classified as more than sixty minutes, thirtyone to sixty minutes, ten to thirty minutes, or**	**Long term, complete or nearly complete pain reduction was accomplished in >80% of patients (with complete follw-up)<br><br>N=6,401 could**	**Level of evidence B because it is a retrospective and not a prospective cohort study.<br><br>This study investigated treatment outcome following total hip arthroplasties**

Reference	Level of evidence	Study type	Number of patients	Patient characteristics	Inclusion criteria	Intervention	Control	Follow-up	Outcome measures	Results	Remarks
				_n= 6467<br>_n= 6458<br>_ THR: 68,6 (range, 24,3-94,8) years<br>_ THR: 66,3 (range, 22,8-94,7) years	osteoarthritis<br>3) ipsilateral involvement of the hip at the time of the primary total hip arthroplasty.<br><br>Patients with unilateral hip disease who had medical comorbidities sufficient to compromise walking capacity were assigned to Charnley class C and therefore were excluded from the study.			4,3 years (range: 29 days-10 years)	less than ten minutes/not possible;<br><u>range of hip flexion</u> was classified as >90°, 71° to 90°, 30° to 70°, or <30°/stiff.<br><br>A modification of “mean age related ability” (MARA) curves was used to show the relationships between preoperative pain and function and postoperative functional outcome.	walk >10 min. preoperative ◊ 57,1% walking capacity >60 min. after 2 years of follow-up.<br>N=6,896 could walk <10 min. preoperative ◊ 38,9% walking capacity > 60 min. after 2 years follow-up<br>Significant difference ($p < 0.01$).<br>All groups showed improvement in walking capacity up to 3-4 years and showed slow but constant improvement thereafter.<br><br>N=10,375 preoperative hip <u>flexion range</u> >70° ◊ 74,7% flexion range >90° at 2 years follow-up.<br>N=2793 preoperative hip flexion range <70° ◊ 62,6% flexion range of >90° at 2 years follow-up (significant difference ($p < 0.01$)).<br>Postoperative improvement pattern and loss	performed with different component designs and fixation modes and included patients from multiple centers and surgeons with different levels of experience. All of these factors could have influenced the study findings.<br><br>Patients usually did not have a complete record of ten documented follow-up examinations and the analysis did not account for clustering of data by center of treatment.<br><br>Thus, the study results may be positively biased by the withdrawal of patients who had an undesired outcome from follow-up routines at the center of the primary intervention.

Reference	Level of evidence	Study type	Number of patients	Patient characteristics	Inclusion criteria	Intervention	Control	Follow-up	Outcome measures	Results	Remarks
										of hip flexion range was equal in all 4 groups.	

Which is the preferred type of prosthesis?

Authors, year	Level of evidence	Study type	Patient characteristic study- versus Control group	Population (incl. sample size)	Inclusion criteria	Intervention	Control	Outcome (effect size, incl. follow-up)	Results	Remarks
Brodner 2003	A2	RCT		100	Unilateral hip arthrosis	Titanium alloclassic stem and cup with M-M bearing	Titanium Alloclassic stem and cup with C-PE bearing	Serum cobalt level for 5 years	M-M group increased serum cobalt levels, C-PE group below detection level	
Bierbaum 2002	B	Multi-center RCT		514		Omnifit HA stem and Ø 32 C-C bearing	Omnifit HA stem and Ø 28 M-PE bearing	4 years follow-up.	Clinical and radiologic Control no difference. No ceramic fracture.	Mediocre support of the follow-up research.
Capello 2008	A2	Multi-center RCT		475		Omnifit HA stem and C-C bearing	Omnifit HA stem and M-PE bearing	Min. 5 years follow-up and 10 years survival data	10 years survival for C-C group is 95,9% and for the M-PE group 91,3%, 0,5% ceramic fracture, equal Harris Hip Score	Authors received financial support of the prothesis manufacturers

Garcia-Rey 2008	A2	RCT		90		Uncemented THA with conventional M-PE bearing	Uncemented THA with cross linked M-HXPE bearing	5 years follow-up,	Mean linear PE erosion a years is 38 _m for the M-PE group and 6 _m for the M-XLPE group	The authors have no conflict of interest
Geerdink 2009	A2	RCT		40	arthrosis	Uncemented THA with conventional M-PE bearing	Uncemented THA with cross linked M-HXPE bearing	Min. follow-up is 7 years	Mean linear PE erosion a year is 142 _m for the M-PE group and 88 _m for the M-XLPE group	
Kim 2005	A2	RCT		104	Age < 50 years and bilateral THA	Uncemented THA with M-HXPE bearing	Uncemented THA with C-HXPE bearing	Mean follow-up is 7 years	Mean linear PE erosion a years is 17 _m for the M-PE group and 8 _m for the C-PE group	
Kraay 2006	A2	RCT		60	Primary THA, age between 50 and 75 years	Cemented stem with metal head and uncemented cup with PE insert	Cemented stem with ceramic head and uncemented cup with PE insert	Mean follow-up is 4 years	Mean linear PE erosion a years is 60 _m for the M-PE group and 55 _m for the C-PE group	Authors doubt if measurements are precise enough
Lewis 2010	A2	RCT		56	Primary THA, age between 18 and 60 years	Uncemented stem with ceramic head and uncemented cup with PE insert	Uncemented stem with ceramic head and uncemented cup with ceramic insert	Mean follow-up is 8 years	Mean linear PE erosion a years is 20 _m for the C-C group and 110 _m for the C-PE group	
Mu 2009	A1	Systematic review of RCT's				7 articles with 8 studies comparing PE with XLPE		Follow-up varies between 2 and 5.5 years	All RCTs show statistically significantly reduced erosion for the XLPE group.	

**McCalden 2009**	**A2**	**RCT**		**100**	**Primary THA, age between 40 and 79 years**	**Hybrid THA with PE insert**	**Hybrid THA with XLPE insert**	**Min. follow-up is 5 years**	**Mean linear PE erosion a years is 51 _m for the M-PE group and 3 _m for the M-XLPE group**	**Authors received financial support of the prothesis manufacturers**
**Seyler 2006**	**B**	**Matched cohort-control**	**M/F 77%/23% Age 45 years**	**79 pt. C-C osteonecrosis 76 pt. C-C arthrosis 26 pt. M-CPE osteonecrosis 25 pt. M-CPE arthrosis**	**Primary THA for osteonecrosis or arthrosis**	**Uncemented ceramic on ceramic THA**	**Uncemented metal on conventional PE THA**	**7 years survival analysis**	**No difference in HHS or survival between ostenecrosis and arthrosis group and no difference between C-C and M-PE group**	
**Rajadhyaksha 2009**	**B**	**Matched cohort-control**	**M/F = 14/11 Mean age 60 resp. 62 years**	**54**	**Primary THA**	**27 uncemented THA's with XLPE insert**	**27 uncemented THA's with PE insert**	**Min. 5 years follow-up.**	**Mean linear PE erosion a years is after imbedding 85 _m for the M-PE group and 22 _m for the M-XLPE group**	
**Triclot 2007**	**B**	**RCT**	**M/F = 50/52 Mean age 71 years**	**102**	**Primary THA**	**Hybrid THA with XLPE insert**	**Hybrid THA with CPE insert**	**Mean 4,9 years follow-up**	**Mean linear PE erosion a years is 106 _m for the M-PE group en25 _m for the M-XLPE group**	

Reference	Level of evidence	Study type	Number of patients	Patient characteristics	Inclusion criteria	Follow-up duration	Outcome measures	Results	Remarks
Eskelinen, 2006<br><br>(77(1): 57-70)	B	Cohort study	8 cup-stem combinations were included	Data from the Finish Arthroplasty Register of 1980-2003<br><br>Cup- stem combinations:<br>1. ABG I/ABG I<br>2. ABG I/ABG II<br>3. Anatomic Mesh/Harris-Galante II<br>4. Biomet Bi-Metric/Mallory<br>5. Biomet Bi-Metric/Romanus<br>6. Biomet Bi-Metric/Universal<br>7. Biomet Bi-Metric/Vision<br>8. PCA Std/PCA Pegged	- Patients <55 years at the time of surgery<br>- Patients with primary arthrosis as indication for surgery<br>- Cup - stem combinations that were used in > 100 surgeries during the study period, including new types with a short follow-up (Mean < 5 years)<br>Excluded:<br>_Uncemented smooth-threaded cups that have well documented bad results.<br>_De Lord Madréporique stem was often used (n = 273, 96%) together with the Lord smooth-threaded cup ◊ this was excluded from the study.	Survival of 5,10,13 years	Survival prosthesis<br><br>The endpoint for survival was defined as revision when either one component or the whole implant was removed or exchanged. Both revision for any reason (including exchange of liner) and aseptic loosening served as endpoints.<br><br>Aseptic loosening was selected as a separate endpoint, because "revision for any reason" also included nonimplantrelated re-operations.	*Revision because of a-septic detachment:*<br>1. ABG I/ABG I<br>Number revisions/ total number of surgeries: 3/105<br>Mean follow-up: 8.2 yrs.<br>At risk (5 yrs.): 99<br>% 5 yrs. Survival (95% CI): 100<br>At risk (10 yrs.): 27<br>% 10 yrs. Survival (95% CI): 60 (91-100)<br>Risk ratio (95% CI): 0.6 (0.2-1.9)<br>*Not statistically significant*<br>2. ABG I/ABG II<br>Number revisions/ total number of surgeries: 3/266<br>Mean follow-up: 4.3 yrs.<br>At risk (5 yrs.): 122<br>% 5 yrs. Survival (95% CI): 99 (97-100)<br>Risk ratio (95% CI): 0.9 (0.3-3.2)<br>*Not statistically significant*<br>3. Anat. Mesh /HG II<br>Number revisions/ total number of surgeries: 14/127<br>Mean follow-up: 9.7 yrs.<br>At risk (5 yrs.): 120<br>% 5 yrs. Survival (95% CI): 98 (95-100)<br>At risk (10 yrs.): 75<br>% 10 yrs. Survival (95% CI): 93 (88-98)<br>At risk (13 yrs.): 20<br>% 13 yrs. Survival (95% CI): 82 (73-92)<br>Risk ratio (95% CI): 1.6 (0.8-3.0)<br>*Not statistically significant*<br>4. Bi-Metric/Mallory<br>Number revisions/ total number of surgeries: 6/107<br>Mean follow-up: 7.5 yrs.<br>At risk (5 yrs.): 95	The risk ratio was adjusted for age and gender. *All types were compared to the Bi–Metric/Universal THR<br><br>*For 'any' revision:*<br>1. ABG I/ABG I<br>Number revisions/ total number of surgeries: 21/105<br>Mean follow-up: 8.2 yrs.<br>At risk (5 yrs.): 99<br>% 5 yrs. Survival (95% CI): 99 (95-100)<br>At risk (10 yrs.): 27<br>% 10 yrs. Survival (95% CI): 79 (70-88)<br>Risk ratio (95% CI): 1.3 (0.8- 2.1)<br>*Not statistically significant*<br>9. ABG I/ABG II<br>Number revisions/ total number of surgeries: 3/266<br>Mean follow-up: 4.3 yrs.<br>At risk (5 yrs.): 122<br>% 5 yrs. Survival (95% CI): 99 (97-100)<br>Risk ratio (95% CI): 0.3 (0.1- 1.0)<br>*Statistically significant (p=0.04)*<br>10. Anat. Mesh /HG II<br>Number revisions/ total number of surgeries: 29/127<br>Mean follow-up: 9.7 yrs.<br>At risk (5 yrs.): 120<br>% 5 yrs. Survival (95% CI): 97 (94-100)<br>At risk (10 yrs.): 76<br>% 10 yrs. Survival (95% CI): 86 (80-93)<br>At risk (13 yrs.): 20<br>% 13 yrs. Survival (95% CI): 63 (51-75)<br>Risk ratio (95% CI): 1.0 (0.7- 1.6)<br>*Not statistically significant*<br>11. Bi-Metric/Mallory<br>Number revisions/ total number of surgeries: 21/107<br>Mean follow-up: 7.5 yrs.<br>At risk (5 yrs.): 96<br>% 5 yrs. Survival (95% CI): 94 (90-99)<br>At risk (10 yrs.): 21<br>% 10 yrs. Survival (95% CI): 62 (46-79)<br>At risk (13 yrs.): 2<br>Risk ratio (95% CI): 1.5 (1.0- 2.5)

Reference	Level of evidence	Study type	Number of patients	Patient characteristics	Inclusion criteria	Follow-up duration	Outcome measures	Results	Remarks
								% 5 yrs. Survival (95% CI): 96 (92-100)<br>At risk (10 yrs.): 20<br>% 10 yrs. Survival (95% CI): 87 (74-100)<br>Risk ratio (95% CI): 1.4 (0.6-3.4)<br>*Not statistically significant*<br>5. Bi-Metric/Romanus<br>Number revisions/ total number of surgeries: 19/106<br>Mean follow-up: 9.4 yrs.<br>At risk (5 yrs.): 99<br>% 5 yrs. Survival (95% CI): 95 (91-99)<br>At risk (10 yrs.): 58<br>% 10 yrs. Survival (95% CI): 86 (78-93)<br>At risk (13 yrs.): 15<br>Risk ratio (95% CI): 2.8 (1.6-4.9)<br>*Statistically significant (p<0.001)*<br><br>6. Bi-Metric/Universal<br>Number revisions/ total number of surgeries: 36/858<br>Mean follow-up: 7.4 yrs.<br>At risk (5 yrs.): 706<br>% 5 yrs. Survival (95% CI): 99 (98-99)<br>At risk (10 yrs.): 216<br>% 10 yrs. Survival (95% CI): 93 (90-96)<br>At risk (13 yrs.): 57<br>% 13 yrs. Survival (95% CI): 89 (85-94)<br>Risk ratio (95% CI): ref.*<br>7. Bi-Metric/Vision<br>Number revisions/ total number of surgeries: 0/385<br>Mean follow-up: 2.6 yrs.<br>At risk (5 yrs.): 55<br>% 5 yrs. Survival (95% CI): 100<br>8. PCA Std/PCA Peg.<br>Number revisions/ total	*Not statistically significant*<br>12. Bi-Metric/Romanus<br>Number revisions/ total number of surgeries: 45/106<br>Mean follow-up: 9.4 yrs.<br>At risk (5 yrs.): 101<br>% 5 yrs. Survival (95% CI): 90 (84-95)<br>At risk (10 yrs.): 60<br>% 10 yrs. Survival (95% CI): 68 (58-77)<br>At risk (13 yrs.): 15<br>Risk ratio (95% CI): 2.2 (1.5- 3.1)<br>*Statistically significant (p<0.001)*<br>13. Bi-Metric/Universal<br>Number revisions/ total number of surgeries: 112/858<br>Mean follow-up: 7.4 yrs.<br>At risk (5 yrs.): 707<br>% 5 yrs. Survival (95% CI): 96 (95-98)<br>At risk (10 yrs.): 220<br>% 10 yrs. Survival (95% CI): 79 (75-83)<br>At risk (13 yrs.): 57<br>% 13 yrs. Survival (95% CI): 74 (69-79)<br>Risk ratio (95% CI): ref.*<br>14. Bi-Metric/Vision<br>Number revisions/ total number of surgeries: 2/385<br>Mean follow-up: 2.6 yrs.<br>At risk (5 yrs.): 55<br>% 5 yrs. Survival (95% CI): 100 (99-100)<br>Risk ratio (95% CI): 0.3 (0.1- 1.1)<br>*Not statistically significant*<br>15. PCA Std/PCA Peg.<br>Number revisions/ total number of surgeries: 40/107<br>Mean follow-up: 11.1 yrs.<br>At risk (5 yrs.): 101<br>% 5 yrs. Survival (95% CI): 95 (91-99)<br>At risk (10 yrs.): 78<br>% 10 yrs. Survival (95% CI): 72 (64-81)<br>At risk (13 yrs.): 40<br>% 13 yrs. Survival (95% CI): 60 (50-70)<br>Risk ratio (95% CI): 1.4 (1.0- 2.1)<br>*Not statistically significant*

Reference	Level of evidence	Study type	Number of patients	Patient characteristics	Inclusion criteria	Follow-up duration	Outcome measures	Results	Remarks
								**number of surgeries: 37/107**<br>**Mean follow-up: 11.1 yrs.**<br>**At risk (5 yrs.): 101**<br>**% 5 yrs. Survival (95% CI): 95 (91-99)**<br>**At risk (10 yrs.): 78**<br>**% 10 yrs. Survival (95% CI): 72 (66-83)**<br>**At risk (13 yrs.): 40**<br>**% 13 yrs. Survival (95% CI): 63 (52-73)**<br>**Risk ratio (95% CI): 4.0 (2.5-6.5)**<br>***Statistically significant (p<0.001)***	
Swedish Hip Arthroplasty Register<br>(Annual Report 2007)	**A2**	Register	**170.413 completely uncemented and cemented hip replacement**		**Total hip prothesis**	**1992-2007**	**Relative risk**	**Uncemented prosthesis implants 33% higher risk compared to cemented prothesis:**<br>**RR= 1.33** ***95% CI: 1.23-1.41***<br>**After 1998 (modern implant design)**<br>**n= 115.959**<br>**RR= 1.37** ***95% CI: 1.13-1.67***<br><br>**Risk early revision (within 2 years)**<br>**Almost double risk for uncemented prothesis implants compared to cemented prothesis**<br>**RR= 1.86** ***95% CI: 1.55-2.23***<br>**Including infections as a risk:**<br>**RR= 2.35** ***95% CI: 1.55-2.89***<br><br>**<u>Uncemented prothesis</u>**<br>**Luxation, loosening, fracture, infection**<br><br>**<u>Cemented prothesis</u>**<br>**Luxation, infection, loosening, fracture**	**Results were only described for the total cemented and uncemented prosthesis; not for the separate components.**

Reference	Level of evidence	Study type	Number of patients	Patient characteristics	Inclusion criteria	Follow-up duration	Outcome measures	Results	Remarks
The Norwegian Arthroplasty Register (Annual Report 2008)	**A2**	Register	**1987-2007 in total 110.985 primary surgeries and 18.496 revisions.**		**Total hip prothesis implants**	**1987-2007**	**Survival**<br>**Relative risk**	**<u>Cemented prothesis</u>**<br>**Years RR *P***<br>**1987-1990 1**<br>**1991-1993 1.6 *<0.001***<br>**1994-1996 1.1 *0.04***<br>**1997-1999 0.8 *<0.001***<br>**2000-2002 0.7 *<0.001***<br>**2003-2007 0.9 *0.1***<br><br>**<u>Uncemented prothesis</u>**<br>**Years RR *P***<br>**1987-1990 1**<br>**1991-1993 0.8 *0.002***<br>**1994-1996 0.5 *<0.001***<br>**1997-1999 0.3 *<0.001***<br>**2000-2002 0.3 *<0.001***<br>**2003-2007 0.9 *0.1***	**RR adjusted for age and sex**
Australian Arthopedic Association (Annual Report 2008)	**A2**	Register	**<u>Cemented prosthesis implants</u>**<br>**Total: 15.864**<br>**Revisions: 380**<br><br>**<u>Uncemented prothesis</u>**<br>**Total: 66.736**<br>**Revisions: 1779**				- **Observed component years (OCY)**<br>- **using the number of revisions per 100 observed component years**<br>- 95% CI<br>- **% revision primary hip replacement**	**<u>Cemented prothesis implants</u>**<br>**OCY: 57.336**<br>**Revision per 100 OCY: 0.7**<br>**95% CI: 0.60-0.73**<br>**% revision after 7 yrs.: 3.8**<br>**95% CI: 3.3-4.3%**<br><br>**<u>Uncemented prothesis</u>**<br>**OCY: 195750**<br>**Revision per 100 OCY: 0.9**<br>**95% CI: 0.87-0.95**<br>**% revision after 7 yrs.: 4.4**<br>**95% CI: 4.1-4.8%**	**The risk of revision depends on patient age.**
National Joint Registry UK (5th Annual Report)	**A2**	Register	**<u>Cemented primary prothesis implants</u>**<br>**Total : 26.685 (43%)**<br>**Of which patient data: 21.810**<br><br>**<u>Uncemented primary prothesis</u>**	**<u>Cemented primary prosthesis implants</u>**<br>**_:**<br>**Tot. 14.337 (66%)**<br>**<45 yrs. 100**<br>**45-54 yrs. 357**<br>**55-64 yrs. 1859**<br>**65-74 yrs. 5282**<br>**75-84 yrs. 5496**<br>**>85 yrs. 1243**<br><br>**_:**<br>**Tot. 7.473 (34%)**		**2003-2007**	- **Revision Rade**<br>- **95% CI**<br>- **Hazard ratio**<br>- **95%CI**<br><br>**For each age category, sex and for cemented and uncemented protheses**	**<u><65 years</u>**<br>**n: 35.288**<br>**RR: 1.8%**<br>**95% CI: 1.6-2.0%**<br>**HR: 1.0**<br>**95% CI: -**<br><br>**<u>65-74 years</u>**<br>**n: 36.881**<br>**RR: 1.0%**<br>**95% CI: 0.9-1.1%**<br>**HR: 0.8**<br>**95% CI: 0.6-0.9**	**There was a decrease in uncemented prosthesis implants of 53% to 43% from 2004 to 2007, respectively. There was an increase in cemented prosthesis implants of 21% in 2004 to 33% in 2007.**

Reference	Level of evidence	Study type	Number of patients	Patient characteristics	Inclusion criteria	Follow-up duration	Outcome measures	Results	Remarks
			**Total : 20.690 (33%)<br>Of which patient data: 16.406**	**<45 yrs. 83<br>45-54 yrs. 244<br>55-64 yrs. 1171<br>65-74 yrs. 3103<br>75-84 yrs. 2445<br>>85 yrs. 427<br><br><u>Uncemented primary prothesis</u><br>_:<br>Tot. 9.398 (57%)<br><45 yrs. 373<br>45-54 yrs. 872<br>55-64 yrs. 2952<br>65-74 yrs. 3341<br>75-84 yrs. 1593<br>>85 yrs. 267<br><br>_:<br>Tot. 7.008 (43%)<br><45 yrs. 411<br>45-54 yrs. 763<br>55-64 yrs. 2239<br>65-74 yrs. 2469<br>75-84 yrs. 1021<br>>85 yrs. 105**				**<u>75+ years</u><br>n: 30.009<br>Revision: 0.9%<br>95% CI: 0.7-1.0%<br>RR: 0.8<br>95% CI: 0.6-0.9<br><br>_<br>n: 41.220<br>RR: 1.4%<br>95% CI: 1.3-1.6%<br>HR: 1.0<br>95% CI: -<br><br>_<br>n: 60.926<br>RR: 1.1%<br>95% CI: 1.0-1.2%<br>HR: 0.9<br>95% CI: 0.8-1.0<br><br><u>Cemented prothesis</u><br>n: 54.769<br>RR: 0.7%<br>95% CI: 0.6-0.8%<br>HR: 1.0<br>95% CI: -<br><br><u>Uncemented prothesis</u><br>n: 28.590<br>Rrevision: 1.8%<br>95% CI: 1.6-2.1%<br>HR: 2.4<br>95% CI: 2.1-2.9**	
Morshed 2007	**B**	SR	**91501 cemented prosthesis implants and 20593 uncemented total hip prosthesis implants**	**20 controlled studies that compared cemented with uncemented total hip prosthesis implants**	**1. All THR's except those placed for a fracture<br>2. controlled comparison of cemented vs. uncemented fixation<br>3. outcome**	**Not specificall y described , only on a per study basis.**	**Survival of the prothesis**	**1. no statistically significant overall difference in survival between the cemented and the uncemented prothesis<br>2. if all ages were studied, than the en cemented prothesis implant had a better survival.<br>3. Cemented Stainless steel or cobalt chrome handles and uncemented titanium handles**	

Reference	Level of evidence	Study type	Number of patients	Patient characteristics	Inclusion criteria	Follow-up duration	Outcome measures	Results	Remarks
					revision for all reasons.<br>4.only randomized trials.			had good results<br>4. For comparisons of cups using a threaded or macro-ingrowth implant with those using a microingrowth or on-growth uncemented design, the former favored cemented fixation whereas the latter did not, and the difference between subgroups was significant.<br>5. As publication year progresses, results of the uncemented prothesis implants improvedr.	
Fitzpatrick, 1998	A1	Health technology assessment<br>SR<br>(no meta-analysis)	11 RCTs were found that compared outcomes of prostheses; Mean n= 186 only 1 RCT with sig. difference	Electronic search Medline and Embase (1980-1995) 11 journals with the highest yieldt of relevant articles	RCT, observational cohort research, observational research with at least 5 years of follow-up	Follow-up mean 3.9 years;	revision percentage	The most favourable revision percentages were found in the Exeter, Lubinus and Charnley prothesis<br>Average results: Muller, McKee-Farrar and Stanmore prothesis.<br>Worst results: Ring, Harris-Galante, PCA and Charnly-Muller prothesis	
Mäkelä, JBJS Am 2008	A2	Register	50968	Group 1 without cement: straight, proximal circumferent porous coated stem and a modulair, porous coated press fit cup<br>Group 2 without cement: anatomically proximal porous coated and/or hydroxyapatite coated stem with a modulair porous coated and/or hydroxyapatite coated pressfit cup<br>Hybrid group :	1. THR,<br>2. > 55 years at the time of surgery<br>3. primary coxarthrosis<br>4. >l 50 implants of prothesis already placed.	0-25 years	Revision for all reasons	With respect to aseptical release<br>1. Uncemented stem prothesis implants better than the cemented stems for patients >74 years. For patients >74 years no difference<br>2. Uncemented cup had a lower revision percentage than the cemented cups for patients <74 years. For patients >74 years the uncemented hydroxyapatite coated press fit cup performed better than cemented cups.<br>3. The cemented prothesis implants had a higher revision percentage after ten years for patients <74 years.	

Reference	Level of evidence	Study type	Number of patients	Patient characteristics	Inclusion criteria	Follow-up duration	Outcome measures	Results	Remarks
				**Cemented stem and pressfit cup**<br>**<u>Cemented group</u>**				**>74 years no differences were observed.**<br>**With respect to revision of the prothesis implants for all reasons: no difference between cemented and uncemented**<br>**Liner change because of polyethylene erosion occurred so often for uncemented prothesis implants for that between group differences disappeared.**	

What is the value of hip resurfacing arthroplasty?

Authors, year	Level of evidence	Study type	Population (incl. sample size)	Inclusion criteria	Intervention (incl. duration, dosage)	Control (incl. duration, dosage)	Outcome (effect size, incl. follow-up)	Results	Remarks
Marker 2009	B	Systematic Review		9 studies RHA versus THA (4 studies RCT and 5 studies cohort)				In general no differences in clinical outcome score.	5 studies positieve RHA for actifity score and change of luxation
Lavigne 2010	A2	RCT	48	Hip arthrosis unilateral, age < 65 yrs,	Resurfacing Hip prothesis (Durom; Zimmer)	Big head circumference THR (CLS stem and Durom head cup; Zimmer)	Walking speed, balance, gait analysis, SF36, WOMAC, UCLA, Merle d'Aubigné	Follow-up 0,3,6 and 12 months. Comparable results for both protheses.	Numbers are low but satisfy the power analysis.
Mont 2009	B	Matched Cohort-control	108	Primary & sec. cox arthrosis. Exclusion: pregnancy, HIV, metal allergy, neurological deficit on the affected leg.	Resurfacing THR (Conserve Plus, Wright Medical)	Regular THR (Osteonics Trident cup and Accolade stem; Stryker)	Primary: HHS, Likert-scale, satisfaction, activity score, complications and X-rays.	Comparable results on effect sizes subject to higher activity scores resurfacing per and postoperative.	Short follow-up (mean 40 months). Power analysis adequate.
Pollard 2006	B	Matched Cohort-control	108	Hip arthrosis unilateral, age < 65 yrs,	Resurfacing THR (BHR; S&N)	Regular hybrid THR	Oxford, UCLA, EuroQol and complications / revisions.	Oxford the same, UCLA and EuroQol higher resurfacing. Revision 6 vs. 8 %.	Follow-up 5-7 years.
Fowble 2009	B	Consecutive Cohort-control	94	Cox arthrosis for self-referred for resurfacing vs. regular THR	Resurfacing THR (Conserve Plus, Wright Medical)	Regular THR (Summit and Pinnacle; DePuy)	HHS, SF-12, UCLA, duration of operation, luxation	Preoperatively statistically significant difference in parameters. HHS the same post-OK versus resurfacing higher SF-12 and UNCLA. Luxation both one.	Follow-up 2-4 yrs. Non-matched control series (demographics different).

**Prosser 2010**	**B**	**Australian Joint Replacement Registry**	**12.093**	**Resurfacing for coxarthrosis September 1999 – December 2008**	**Resurfacing THR**	**Regular THR in similar period**	**Revision operation**	**Statistically significant more revisions with hip circumference < 50 mm. After 8 yrs revisions RHP > THR (5.3 vs. 4.0 %). Pat < 55 yrs and hip head circumference >50 mm revision after 7 yrs RHP = 3.0 %. Revision percentages differ for per design.**	

Which is the preferred surgical approach for total hip replacement?

Reference	Level of evidence	Study type	Number of patients	Patient characteristics	Inclusion criteria	Intervention	Control	Follow-up duration	Outcome measures	Results	Remarks
**Jolles and Bogoch, 2009**	**B**	Systematic review of 4 prospective cohort studies	241 patients and prothesis	**Primary total hip prothesis**	**RCT**	**Total hip prothesis at posterolateral or direct lateral approach**	**No**	**?**	**Luxation Trendelenburg gait Nerve damage Pain**	**No differences in luxation were observed between posterolaterel and the direct lateral approach. The same for trendelenburg gait Direct lateral approach results in more nerve damage (not n.ischiadicus) End rotation movement is greater for the posterolateral approach**	**Few studies satisfied criteria, therefore no conclusion could be made.**
**Kwon *et al,* 2006**	**B**	**Systematic review of 1 RCT and cohort studies**	**4115 prothesis**	**Primary total hip prothesis**	**Studies that described approach and recovery of the protective casin**	**Total hip prothesis via the posterolateral with (PL+) and without recovery of the protective casin (PL-)**	**Studies that described luxation for other approaches**	**Minimum 6 months**	**Luxation**	**PL+: 1648 THR – 8 luxation (0,49%) PL-: 2467 TPH– 110 luxation (4,49%) (RR 8,21;95%CI 4,05-16,67) Anterolateral: 2147 THR – 15 luxation (0,70%) Lateral: 2309 THR– 10 luxation (0,43%)**	**Type of prothesis? Experience? Position prothesis?**
**Masonis *et al,* 2002**	**B**	Systematic review of cohort studies	13233 prothesis implants with respect to luxation<br><br>2455 prothesis implants with respect to limping	**Primary total hip prothesis**	**Studies that described luxation and limping**	**Luxation and limping**	**None**	**?**	**Luxation Limping**	**Trans troch: 2988 – 38 lux. (1,27%) PL+: 2262 – 46 lux (2,03%) PL- :3719 – 141 (3,95%) Anterolateral: 826 – 18 lux (2,18%) Direct lateral: 3438 – 19 lux (0,55%)<br><br>Anterolateral – lateral approach limping: 4-20% Posterolateral approach limping: 0 – 16 %**	**Little literature about limping and luxation.<br><br>The available literature did not allow for meta-analysis**

What is the value of minimal invasive surgery?

Reference	Level of evidence	Study type	number of patients	Patient characteristics	Inclusion criteria	Intervention	Control	Duration of follow-up	Outcome measures	Results	Remarks
**Verteuil *et al.*, 2008**	A1	Systematic review	12 RCTs<br><br>22 observational studies<br><br>8 case series<br><br>1 registration (Norway)	**RCT: majority < 200 patients (20-219)**<br><br>**<u>Single mini-incision THR (9 RCTs):</u>**<br>**SI:**<br>**n= 492**<br>**Mean age : 65,7 yrs.**<br>**MI:**<br>**n= 487**<br>**Mean age: 64,8 yrs.**<br><br>**<u>Two mini-incision THR (3 RCTs):</u>**<br>**Two:**<br>**n= 63,3**<br>**Mean age: 65,7 yrs.**<br>**MI:**<br>**n= 63,3**<br>**Mean age: 64,8 yrs.**	**Long term follow-up (revision data) from registry (Norway).**<br><br>**Article in English, Chinese or Japanese.**<br><br>**THR with respect to arthritis.**<br><br>**<u>Excluded:</u>**<br>**Article focused on THR for reasons such as, osteoporosis, fractures or tumors. Revision surgery, resurfacing or computer modeling Surgery.**	**<u>9 RCTs</u>**<br>**Single mini-incision THR**<br><br>**<u>2 RCT</u>**<br>**Two mini-incision THR**<br><br>**<u>1 RCT</u>**<br>**Two mini-incision THR**	**Standard THR**<br><br>**Single mini-incision THR**<br><br>**Standard THR**	**<u>5 RCTs</u>**<br>**less than a year.**<br><br>**minimum 1 year (case series or cohort study with two or more surgeons)**<br><br>**at least 3 years (case series with 1 surgeon)**	(only RCTs)<br><br>MI=mini-incision vs. S=standard<br><br>Revision (n; MI: 197; S:198)<br><br>**Postoperative luxation**<br>(n; MI: 347; S: 352)<br><br>**Implant position (cup)**<br>(n; MI: 235; S: 239)<br><br>**Implant position (stem)**<br>(n; MI: 323; S: 331)<br><br>**Infection**<br>(n; MI: 407; S: 412)<br><br>**Deep venous thrombosis**<br>(n; MI: 317; S: 322)<br><br>**Operation duration**<br>(n; MI: 427; S: 432)<br><br>**Blood loss**<br>(n; MI: 347; S: 352)<br><br>**Hospital stay**<br>(n; MI: 297; S: 302)<br><br>**Harris hip score (≤ 3 months)(n; MI: 167; S: 168)**<br>**Harris hip score (> 3 months)(n; MI: 217; S: 219)**	**Peto OR (95% CI)**<br><br>**7,96 (0,16 – 402) (*p*= 0,30) compared to. standard**<br>**1,72 (0,43 – 6,92) (*p*= 0,45) t.g.v. standard**<br><br>**0,93 (0,50 – 1,74) (*p*= 0,83) compared to MI**<br><br>**0,70 (0,35 – 1,40) (*p*= 0,45) compared to MI**<br><br>**7,48 (0,78 – 72,16)(*p*= 0,08) compared to standard**<br><br>**0,39 (0,12 – 1,30) (*p*= 0,12) compared to MI**<br><br>**WMD -3,70 (-5,67-1,74) (*p*= 0,0002) compared to MI**<br>**WMD -56,59 (-71,63- -41,55) (*p*< 0,00001) compared to MI**<br>**WMD -0,50 (-0,83- -0,18) (*p*= 0,002) compared to MI**<br>**WMD -1,25 (-3,75-1,24) (*p*= 0,33) compared to MI**<br>**WMD 0,35 (-0,13-0,83) (*p*= 0,152) compared to.**	**Included cost effect analysis:**<br>**Mean QUALY with 1 years: 0,677 standard THR**<br>**0,695 mini-incision THR**<br>**Mean QUALY with 40 years:**<br>**8,463 standard THR**<br>**8,480 mini-incision THR**<br>**(only fot outcomes revision, postoperative luxation, deep venous thrombosis and pulmonary embolia)**<br><br>**Too little standardization in measurement of outcome measures**<br><br>**Reviewers were not blinded for author, institute or publication details.**

Reference	Level of evidence	Study type	number of patients	Patient characteristics	Inclusion criteria	Intervention	Control	Duration of follow-up	Outcome measures	Results	Remarks
										standard	
Wall *et al.,* 2008	B	Review	**69 studies of which 9 RCTs**	**Not described**	**Studies in English language published between 1998 and 2008**	**minimal invasive surgery (no 2-insicion approach)**	**Standard approach (posterior and lateral)**	**< 2 years**	(only RCT)<br><br>***Ogonda et al., 2005***<br>**Bloedverlies**<br>***Bennett et al., 2006/ Lawlor et al., 2005***<br>***Dorr et al., 2007***<br><br>***Kim et al., 2006***<br><br><br><br><br><br><br>***Chimento et al., 2005***<br><br><br><br><br><br><br>***Pour et al., 2007***<br><br><br><br><br><br>***Duka et al, 2007***<br><br><br><br>***Hart et al., 2005***	**Statistically significant difference (52 ml difference)**<br>**No statistically significant differences**<br><br>**MIS group had shorter Hospital stay, faster mobility and less pain.**<br>**MIS group had statistically significant shorter time of surgery and fewer total blood replacement.**<br>**MIS group had statistically significantly less blood loss (Mean difference 43 ml) and that fewer patients limped after 6 weeks.**<br>**The treatment of patients had a statistically significant greater effect on the outcomes than cut length.**<br>**MIS group had statistically significant shorter operation time and blood loss.**<br>**The early Harris Hip score was better in the standard group.**<br>**No statistically**	**They searched only in Pubmed.**<br><br>**3 of the 9 RCTs were from the same research group . (*Ogonda et al., 2005; Bennett et al., 2006; Lawlor et al., 2005*)**<br><br>**No pooled results**<br><br>**Quality of the included studies was determined using the Cochrane Reporting Quality Score ◊ 12 criteria, score of 0-2 (Total 24 points)**<br>**Range score review 12- 22 points.**

Reference	Level of evidence	Study type	number of patients	Patient characteristics	Inclusion criteria	Intervention	Control	Duration of follow-up	Outcome measures	Results	Remarks
										**significant difference.**	
**Goldstein *et al.*, 2008**	B	Comparative study	538 total hip prothesis.<br><br>221 x THR via MIS<br><br>317 x via standard<br><br>512 patients with a min follow-up of 1 years were given a questionnaire to evaluate the cosmic results of the surgery.<br><br>287 patients returned the questionnaire; 123 hips via MIS for 109 patients and 186 hips via the standard procedure in 171 patients.	**<u>MIS group (n hips= 123)</u>**<br>**Incision ≤ 5 inches**<br>**Mean follow-up: 21 months (12-36)**<br>**_/_: 62/61**<br><br>**<u>Standard group (n hips = 186)</u>**<br>**> 5 inches**<br>**Mean follow-up: 25 months (12-36)**<br>**_/_: 111/75**	**A primary total hip prothesis between March 2001 and March 2003.**	**Questionnaire after >1 years to evaluate the cosmic results of the surgery.**	**n.a.**	**> 1 years.**	-Opinion about the cosmetic appearance of the scar.<br><br>- uneven scar edges<br><br>-Is the skin next to the scar wrinkled or bumpy?<br><br>- is the space between the edges of the scar too big?<br><br>- do the edges of the scar curl or sink?<br><br>-is the scar swollen or thick?	**95% of both groups reported that the scar looked good.**<br>**In the MIS group was the percentage higher of patients that reported uneven scar edges and a wrinkled/bumpy skin around the scar.**<br>**Standard group : 12 patients**<br>**MIS: 5 patients space between the edges too big. Difference NS (*p*-0.451)**<br>**MIS group sig. Higher number of incidents of sinking or curling of the edges of the scar compared to the standard group (*p*= 0,001)**<br>**MIS: 2 patients Standard: 1 patient reported a swollen scar.**<br><br>**<u>Standard group :</u>**<br>**72% of de patients scored the optimum (of 6) for cosmic outcomes > 1 yrs. follow-up postoperative.**<br>**<u>MIS:</u>**<br>**64% of de patients scored the**	**This is a subjective measurement.**<br><br>**Selection procedure was not clearly described.**

Reference	Level of evidence	Study type	number of patients	Patient characteristics	Inclusion criteria	Intervention	Control	Duration of follow-up	Outcome measures	Results	Remarks
										**optimum (of 6) for comic outcomes > 1 yrs. follow-up postoperative.**<br><br>**No complications were reported.**	
**Mahmood *et al.*, 2007**	C	Review	36 articles of:<br><br>3 RCTs *(were also included in Verteuil et al., 2008)*<br><br>6 observational studies (prospective)<br><br>8 cohort studies<br><br>9 retrospective studies<br><br>10 case series<br><br>6098 patients; 6626 THR<br><br>5285 patients with I<br><br>Of which 1341 THR SMI	**<u>Intervention</u><br>Mean 62,2 yrs. (48-73,4)<br>Mean BMI: 26,7 (25 studies)<br><br><u>Control</u><br>Mean 63,3 yrs. (49-69)<br>Mean BMI: 28,2 (25 studies)**	**Only published papers in the English language in peer-reviewed journals.**	minimal invasive incision procedure (MI)	**Single incision procedure (SI)**	**65,1 weeks (range: 4-260 weeks)**	Blood loss<br><br>Average operation time<br><br>Average Hospital stay<br><br><u>Complications</u><br>Infection<br>Luxation<br>Thrombosis<br>Early revision<br>Intra-operative fracture<br>Nerve damage	**NS average difference in blood loss reported in 18 of the 36 studies.<br>MI: 80,4 min. (range 37,5-148 min.)<br>SI: 86,5 min. (range 54-166 min.) NS<br>MI: 3,69 days (range 1-6 days)<br>SI: 4,98 days (range 3-6 days) (*p= 0,024*)<br><br>NS<br>NS<br>NS<br>NS<br>NS<br>NS**	**Methodologically scored using the Coleman score. Scored by two researchers. Scored using 10 criteria on a scale of 0-100 (with 100 representing very good quality)<br>Mean score: 48,2 (range 27-82)<br>5 criteria were weakly present. Type of study, description of rehabilitation protocol, outcome criteria, outcome estimates, process of subject selection.<br><br><u>Limitation studies:</u><br>Only studies published in the English language were included. Comparison of outcomes was simplistic (reported averages were compared although there was a lot of heterogeneity in study design)<br>This reviews does not describe which studies were included.**

Reference	Level of evidence	Study type	number of patients	Patient characteristics	Inclusion criteria	Intervention	Control	Duration of follow-up	Outcome measures	Results	Remarks
											**The search strategy was not clearly described.**
**Chen *et al.*, 2009**	B	Prospective, randomized	83 in group 1 and 83 in group 2	**Group 1: minimal invasive two incision technique transgluteal Group 2: traditional transgluteal approach**	**Patients with coxarthrosis; similar prothesis for both groups**	Minimal invasive 2 incision approach	**Traditional transgluteal approach**	**2 years**	Operation duration, blood loss, technical problems, cup inclination, ante version angle, stem alignment, canal filling ratio, Harris Hip Score, WOMAC, pain relieve.	**Group 1: longer duration of operations, more blood loss, more complications Group 1: passagère laesie N.cut.fem.lat 27 (32,5%) proximal femur fracture 6 (7.2%) area wound infection 1 (1,2%) group 2: proximal femur fracture 4 (4,8%) area wound infection 1 (1,2%) luxation 1 (1,2%) less NSAIDs used in group 1 and for a shorter period**	
**Nuelle *et al.*, 2007**	B	Prospective	50 patients in each group	**Group 1: 11 hip prothesis, 14 knee prothesis Group 2: 8 hip prothesis, 17 knee prothesis All approaches were normal traditional**	**Hip- and knee prothesis.**	Group 2 this program is normally offered to patients given a minimal invasive treatment (mini protocol)	Group 1 normal program of anesthesia, postoperative pain relieve and physiotherapy	**Not described**	ADL tests, duration of hospital stay	**Group 2 (mini protocol) quicker recovery**	
**Mow *et al.*, 2005**	B	Prospective	Group 1: 20 patients<br>Group 2: 14	**Group 1: mini-posterior Group 2: standard posterior approach**	**Hip prothesis**	All scars were photographed, length of scar was immeasurable on the photo. Two researchers judged the photos independently using similar criteria.<br>Patient opinion	2 researchers	**17 months**	Scar: color, contour, deformation, Fitzpatrick classification, general appearance	**Scars after the standard posterior approach were prettier than after a mini-posterior approach**	**N= Small Were the groups comparable?**

Reference	Level of evidence	Study type	number of patients	Patient characteristics	Inclusion criteria	Intervention	Control	Duration of follow-up	Outcome measures	Results	Remarks
						was also assessed.					
**Mardones et al., 2005**	C	Corpse study	10 corpses	**Each corpse obtained a prothesis one side placed using the 2-incision technique and a prothesis on the other side placed using the mini-posterior approach**	**Ad random**	Measurement of muscle tissue damage.	no	**no**	Measurement of length and breadth of the muscle damage	**Both approaches damaged the muscles and this damage was more pronounced at the 2 incision technique.**	**No comparative study; case series N= small**

Which prophylactic measures for infection should be applied in primary total hip replacement?

Reference	Level of evidence	Study type	number of patients	Inclusion criteria	Patient characteristics	Intervention	Control	Follow-up	Outcome measures	Results	Remarks
**Albuhairan, 2008**	A1	meta-analysis or 26 RCTs	11.343<br><br>(26 RCTs)	**- Patients with primary or revision THR<br>- antibiotics preop administered<br>- Reporting outcome measure wound infection<br>- study type RCT**	**Not reported**	**1) antibiotics (AB)<br>2) systemic AB<br><br><br>3) teicoplanin<br><br><br>4) first generation cephalosporin<br>5) all generation cephalosporin**	**1) no AB<br>2) with antibiotic-impregnated cement<br>3) first and second generation cephalosporin<br>4) second generation cephalosporin<br>5) penicillin derivate**	**From 10 days to 10 years**	**Wound infection defined as: visible purulent exudate at the surgical site**	**<u>Stat. sign.:</u><br>AB vs. no AB:<br>N=3065 (7 RCTs)<br>RR 0,19 (95%BI 0,12-0,31)<br><br><u>NS:</u><br>Syst. AB vs. with AB-impregnated cement:<br>N=2388 (3 RCTs)<br>RR 0,88 (95%BI 0,59-1,31)<br><br>Teicoplanin vs. cephalosporin:<br>N=2625 (5 RCTs)<br>RR 1,22 (95%BI 0,64-2,34)<br><br>first gen vs. second gen cephalosporin:<br>N=2879 (8 RCTs)<br>RR 1,08 (95%BI 0,63-1,84)<br><br>Cephalosporin vs. penicillin derivate:<br>N=386 (3 RCTs)<br>RR 1,17 (95%BI 0,31-4,41)**	**Adequate randomisation(+/-/?): + (5 RCTs), ? (21 RCTs)<br><br>Allocation concealment (+/-/?): + (4 RCTs), ? (22 RCTs)<br><br>Blinding outcome assessor: triple (1 RCT), double (7 RCTs), single (6 RCTs), ? (12 RCTs)<br><br>Intervention- and control group control group comparable (+/-/?): [illegible] (17 RCTs), ? (9 RCTs)<br><br>Sufficient follow-up (≥80%) (+/-/?):<br>+ (12 RCTs), ? (11 RCTs), - (3 RCTs)<br><br>Intention-to-treat analysis (+/-/?):<br>+ (4 RCTs), ? (10 RCTs), - (12 RCTs)<br><br>Financing:<br>No financial suppor[illegible] from commercial party.**

Reference	Level of evidence	Study type	number of patients	Inclusion criteria	Patient characteristics	Intervention	Control	Follow-up	Outcome measures	Results	Remarks
**Gillespie, 2010**	A1	meta-analysis of 23 RCTs	N=8447 (23 RCTs)	**- Patients who undergo internal fixation or revision arthroplasty for closed fracture of proximal femur or other long bone. - antibiotics preop administered<br>- Reporting outcome measure wound infection<br>- study type RCT**	**Not reported**	**1) 1 preop doses and ≥2 postop doses parenteral antibiotics (AB)<br><br>2) 1 preop doses parenteral AB<br><br>3) 1 doses parenteral AB<br><br>4) 1 doses parenteral AB with long half-time<br><br>5) earlier multiple doses AB administered in ≤24 h<br><br>6) oral administration of AB**	**1) placebo or no treatment<br><br>2) placebo or no treatment<br><br>3) earlier multiple doses similar AB<br><br>4) earlier multiple doses AB with shorter half-time<br><br>5) earlier multiple doses AB administered in >24 h<br><br>6) parenteral administered AB**	**Not reported**	**Wound infection defined as:<br>- deep wound infection (DWI) : occurrence < 1 year postop, implant in right position, infection affects tissue underneath fascie infection<br>- Superficial wound infection (SWI): occurrence < 30 days postop, affects subcutaneous skin tissue or muscles superior of fascie.<br><br>Other infections (urine tract (UWI), airway (AWI))**	**<u>Stat. sign.:</u><br>1): N=1915 (10 RCTs)<br>DWI: RR 0,35 (95%BI 0,19-0,62)<br>SWI: RR 0,38 (95%BI 0,22-0,66)<br>UWI: RR 0,63 (95%BI 0,53-0,76)<br>AWI: RR 0,46 (95%BI 0,33-0,65)<br><br>2): N=3500 (7 RCTs)<br>DWI: RR 0,40 (95%BI 0,24-0,67)<br>SWI: RR 0,69 (95%BI 0,50-0,95)<br><br><u>NS.:</u><br>Comparison3-6**	**Adequate randomisation(+/-/?): + (5 RCTs), ? (1[illegible] RCTs), - (3 RCTs)<br><br>Allocation concealment (+/-/?): + (7 RCTs), ? (13RCTs), - (3 RCTs[illegible]<br><br>Blinding outcome assessor (+/-/?):+ (8 RCTs), ? (9 RCTs), - (6 RCTs)<br><br>Intervention- and control group control group comparable (+/-/?): Not reported<br><br>Sufficient follow-up (≥80%) (+/-/?): + (4 RCTs), ? (12 RCTs), - (7 RCTs)<br><br>Intention-to-treat analysis (+/-/?): Not reported<br><br>Financing: No conflict of interest**
**Engesaeter, 2003**	B	Retrospective (register) study: effectiveness AB prophylaxis is on revision percentage	N=22.170 THR	**- Patients with implants and cement and available long term Results in registry.<br>- primary implants in patients met idiopathic hip osteoarthritis<br>- 1 of 4 cemented**	**71% F<br>Mean age: 72 (17-97)**	**Cemented implants**	**n.a.**	**0-14 years postop**	**revision percentage**	**696/22.170 revisions (3.1%), of which 440/696 (2.0%) because of aseptic loosening and 102/696 (0.5%) deep infection.<br><br>Chance of revision syst+cement vs. syst alone:<br>RR 1.4 (95%BI 1,1-1,7).**	**Selective loss to follow-up (+/-/?): ?<br><br>Sufficient follow-up (+/-/?): +<br><br>Financing: No conflict of interest**

Reference	Level of evidence	Study type	number of patients	Inclusion criteria	Patient characteristics	Intervention	Control	Follow-up	Outcome measures	Results	Remarks
				**cup/stem implants combinations:: Charnley/Charnley (DePuy, Leeds, UK), Exeter/Exeter (Howmedica International, Herouville, France), Titan/Titan (DePuy, Chaumont, France) or Spectron/ International total Hip (ITH) (Smith & Nephew, Memphis Memphis, Tennessee).<br>- implant with high-viscosity cement Palacos with or without gentamicin (Schering-Plough International Inc., Kenilworth, New Jersey) or Simplex m/z colistin/erythromycin (Howmedica International, London, UK).<br>- AB prophylaxis with cephalosporin (first gen. cephalotin or second gen. cefuroxime) or penicillin (cloxacillin or dicloxacillin, both semi synthetic penicillinase-resistent)**						**AB prophylaxis regime with syst+cement: 4x daily at day of operation sign. better result than ≤3xdaags**	
**Parvizi, 2008**	B	meta-analysis of 6 comparati	N=24.661 hip replace ments	**- Patients who undergo primary or revision THR**	**Not reported**	**with AB-impregnated cement**	**cement without AB**	**Not reported**	**- deep wound infection (DWI)**	**<u>Stat. sign.:</u><br>N=15.137 primary THR (6 studies)**	**Meta-analysis reports no information about**

Reference	Level of evidence	Study type	number of patients	Inclusion criteria	Patient characteristics	Intervention	Control	Follow-up	Outcome measures	Results	Remarks
		comparative studies<br><br>(meta-analysis of studies, mostly level B)	ments (6 studies)<br><br>N=21.445 analysed	**- Reporting outcome measure deep wound infection and overall survival<br>- study type comparative study with AB-impregnated cement vs. cement without AB<br><br>Exclusion:<br>- 'Boneloc' cement, Simplex cement**					**- overall survival (OS)**	**DWI: RR 0,51 (95%BI 0,34-0,75)<br><br>N=55.600 revision THR (5 studies)<br>DWI: RR 0,72 (95%BI 0,63-0,83)<br><br>Survival:<br>Prim. THR: 98%<br>Rev. THR: 88%**	**the included studies (except for being comparative). The authors state to have done a quality assessment.<br>Financing: unknown**
**Lidwell, 1982**	B	Multicenter RCT (19 centres in England, Scotland and Sweden)<br><br>Study period:1974-1979	**N= 8136 surgeries<br>N=8055 analysed (6781 hip 1274 knee)**	**- Patients who undergo THR or knee replacement**	**Not reported**	**OR with ultraclean air (UCA)(<10KVE/m^3) ventilation system; yes/no use whole-body exhaust-ventilated suits<br>NB: hospitals used different UCA systems => different levels of contamination**	**OK with conventional ventilation system (modern, positive air pressure)<br>NB: large variation in median KVE/m^3**	**Mean. duration follow-up: 2-2,5 jr**	**Deep infection, defined as bacterial joint infection with associated with clinically apparent tissue damage.**	**Infection percentage I: 23 / 3922 = 0,57%<br>C: 63 / 4133 = 1,5%<br>I vs. C:<br>RR 0,38 (95%BI 0,24-0,62)**	**Adequate randomisation(+/-/?): ? no uniform randomisation method<br><br>Allocation concealment (+/-/?): ? (unplanned alternations in randomisation list, in one hospital _ of all surgeries was in control environment)<br><br>Blinding outcome assessor (+/-/?): -<br><br>Intervention- and control group control group comparable (+/-/?): [illegible]<br><br>Sufficient follow-up (≥80%) (+/-/?): + (1% [illegible] no reasons reported)<br><br>Intention-to-treat analysis (+/-/?): -**

Reference	Level of evidence	Study type	number of patients	Inclusion criteria	Patient characteristics	Intervention	Control	Follow-up	Outcome measures	Results	Remarks
											**Substantial variatio[illegible] in use of AB prophylaxis.**<br><br>**Financing: unknown**
**Persson, 1999**	B	Cost-effectiveness study	Whole register ?	**Patients from the Swedish arthroplasty register who received antibiotics.**	**Swedish arthroplasty register**	**Price and risk of aseptic loosening**	**n.a.**	**n.a.**	**Relation between risk of aseptic loosening and costs**	**Plain Palacos results in best price/quality ratio**<br>**Sulfix, Simplex and CMW have a higher risk of aseptic loosening at higher cost than plain Palacos**<br><br>**Palacos gentamicine gives a lower risk of aseptic loosening, but at considerable higher cost.**<br><br>**Keeping in mind the reduction of the risk of deep infection is a combination of systemic antibiotics with gentamicine cement and 'surgical enclosure' the most cost-effective method.**	
**Meehan, 2009**	**D**	current concepts review, no primary study of meta-analysis									
**Kasteren, 2007**	C	Retrospective cohort study	1922	**Surgical prophylaxis and Surveillance. (CHIPS) project**<br><br>**2000–2002**<br>**11 v.d.13 Dutch hospitals involved in CHIPS project provided data on primary THR**<br><br>**postoperative wound infection according to US**	**Female: 69%**<br>**Mean age (±SD) 68,8±10,8 years**<br>**ASA score: >2 ◊ 12%**<br>**Mean stay preop: (±SD) 1,2±2,1 days**<br>**Mean. duration procedure: (±SD) 78,.6±35, 3 min,**<br>**Mean. hospital stay postop: (±SD) 8,8±5,6 days**	**n.a.**	**n.a.**	**n.a.**	**Risk factors for postoperative wound infection after THR**	<u>**Antibiotics prophylaxis**</u><br><br>***OR(95% CI)***<br>**Prophylaxis duration**<br>Single doses<br>**= Reference**<br><br>**Multiple postoperative doses ≤ 24 hour**<br>**2,0 (0,6–7,0) NS**<br>**Multiple postop doses >24 hours**<br>**1,4 (0,2–9,2) NS**<br>**Administration of prophylaxis 160 min before incision**<br>**1,3 (0,4–4,4) NS**	**All patients receive[illegible] antimicrobial prophylaxis.**<br><br>**The used antibiotic[illegible] were classified according to the Dutch group for antibiotics-policy clinical guideline. (cefazolin [*n*=947], flucloxacillin [*n*=48][illegible] and erythromycin [*n*=8] or clindamyci[illegible] [*n*=1] in case of**

Reference	Level of evidence	Study type	number of patients	Inclusion criteria	Patient characteristics	Intervention	Control	Follow-up	Outcome measures	Results	Remarks
				**Centres for Disease control and Prevention-criteria.**						**31–60 min before incision 0,9 (0,4–2,1) NS 1–30 min before incision ◊ Reference During or after incision 2,8 (0,9–8,6) NS Administering of AB-impregnated cement 0,8 (0,3–1,9) NS Patient- and procedure related variables Age, year 1,4 (1,0–2,1) NS Female gender. 1,7 (0,7–3,9) NS ASA score 1◊ Reference 2◊ 1,5 (0,6–3,8) NS 3+◊ 2,8 (0,8–9,2) NS Operation duration in the 175th percentile 2,5 (1,1–5,8) P= 0,04**	**allergy) or with broad spectrum (cefamandole [*n*=39], cefuroxime [*n*=873], amoxicillin plus netilmicin [*n*=1], and clindamycin plus gentamicin [*n*=1]).**
**Bowers, 1973**	C	Animalstudy medication stage I-III	80	**healthy canines ≥ 18kg, negative blood analysis**	**healthy canines ≥ 18kg, negative blood analysis**	**Antistaphylococce treatment**	**?**	**?**	**(1) permeability of antibiotics and persistence in the bone (2) effect of cephaloridine administration in standard wound infection**	**Cephaloridine easily penetrated hematomas in the bone and persisted bacteriological concentrations. Preop cephaloridine resulted in sterile wounds that did not infect. Cephaloridine administered ≥6 hour after: infection stable.**	
**Classen, 1992**	A2	Prospective cohort study	2847	**Patients planned for surgery May 1985-November 1986<br><br>Exclusion:<br>-Surgery within >48h after hospital admission<br>-Patients who**	**Mean age. 53 years (range, 11-97)<br>Female:<br>n= 1758<br>Male:<br>n= 1089<br>Mean hospital stay: 7,6 days<br><br>55 patients died**	**<u>Early administration</u> n= 369<br>(2-24 hours before incision)<br><br><u>Preoperative</u><br>n= 1708<br>(2 hours before incision)**	**n.a.**	**?**	**Surgical wound infection**	**<u>Early administration</u><br>% infections: 3,8<br>RR: 6,7<br>95% CI: 2,9-14,7<br>OR: 4,3<br>95% CI: 1,8-10,4<br><br><u>Preoperative</u><br>% infections: 0,59<br>RR: 1**	

Reference	Level of evidence	Study type	number of patients	Inclusion criteria	Patient characteristics	Intervention	Control	Follow-up	Outcome measures	Results	Remarks
				**received no antibiotics<br>-treatment with antibiotics > 28 before or after surgery<br>-Patients who had an existing infection<br>-surgery for which antibiotics are not recommended-Patients who had more than 1 surgery during the same hospital stay.**	**during hospital admission<br><br>1359 clean operations<br>1488 clean-contaminated operations**	**Perioperative<br>n= 282<br>(during 3hours after incision)<br><br>Postoperative<br>n= 488<br>(between 3 and 24 hours after incision)**				**Perioperative<br>% infections: 1,4<br>RR: 2,4<br>95% CI: 0,9-7,9<br>OR: 2,1<br>95% CI: 0,6-7,4<br><br>Postoperative<br>% infections: 3,3<br>RR: 5,8<br>95% CI: 2,6-12,3<br>OR: 5,8<br>95% CI: 2,4-13,8<br><br>A stepwise logistic regression shows that preoperative administration of antibiotics results in the lowest risk of postoperative wound infection.**	
**Stefansdotti, 2009**	C	Retrospective cohort study	114	**?**	**Group 1 N= 114 university clinic in Lund 2008<br><br>Group 2 N= 291 patients from Swedish knee arthorplasty register**	**n.a.**	**n.a.**	**-**	**Time of administrating antibiotics before surgery**	**Group 1<br>n=51 received first doses antibiotics 15-45 min before surgery.<br>N=22 surgery had started or antibiotics were administered at the start of surgery<br><br>Group 2<br>N= 113 received first doses antibiotics 15-45 min before surgery.**	**Care provider was blinded**
**Streinberg, 2008**	A2	Prospective cohort study	4472	**July –November 2003 (baseline)<br>February-July 2005 (measurement)<br><br>Surgeries hart patients (n=1949), hip and knee implants (n=1735) and hysterectomy (n=788).**	**N=3405; cephalosporin alone or antibiotics designed by SCIP (surgical care improvement project) added within 60 minutes before incision,<br>N= 575; Cephalosporin plus vancomycin,<br>N= 218; vancomycin alone,**	**Group 1 N=1844<br>Vancomycin/Fluoroquinolones added within60 minutes or cephalosporin added within 30 minutes before incision<br><br>Group 2 N= 1796<br>Vancomycin/Fluoroquinolones 61-120 minutes or**	**Group 4 N=188<br>Port-incision**	**-**	**Time of administration of prophylaxis to prevent postoperative wound infection**	**113 infections in 109 patients<br><br>Group 1<br>Infection risk:<br>2,1 %<br>RR (95% CI): Reference<br>OR (95% CI):<br>Reference<br>Group 2<br>Infection risk:<br>2,4 %<br>RR (95% CI): 1,16 (0,75-1,79)<br>OR (95% CI):<br>1,48 (0,92-2,38)**	

Reference	Level of evidence	Study type	number of patients	Inclusion criteria	Patient characteristics	Intervention	Control	Follow-up	Outcome measures	Results	Remarks
					N= 240;<br>Fluoroquinolones with or without agents,<br>N= 34; antibiotics (not documented)	cephalosporin 31-60 minutes before incision<br><br>Group 3 N= 644<br>other pre-incision supplement				Group 3<br>Infection risk:<br>2,8 %<br>RR (95% CI): 1,36 (0,78-2,36)<br>OR (95% CI):<br>1,30 (0,70-2,41)<br>Group 4<br>Infection risk:<br>5,3 %<br>RR (95% CI): 2,58 (1,31-5,10)<br>OR (95% CI):<br>2,20 (1,03-4,66)	
Soriano, 2006	B	Prospective cohort study	Period A<br>n= 256<br><br>Period B<br>n= 256	-Patients who underwent surgery for 'femoral neck fracture'<br><br>Period A<br>January - May 2002<br><br>Period B<br>June 2002 - May 2003	Period A<br>Mean age(years): 80,1 ± 10,1<br>Female/male 4,8/1<br><br>Period B<br>Mean age (years): 81,6 ± 9<br>Female/male<br>5/1	Period A<br>2 doses 1,5 g cefuroxime, 1 during de anaesthesia introduction and the other 2 hours postop.	Period B<br>cefuroxime plus 600 mg teicoplanin during de anaesthesia introduction	12 months	Incidence of postoperative wound infection	Period A<br>total % infections 5,07% (n=13 of 256)<br>N= 7 ◊ MRSA<br><br>Period B<br>total % infection2,36% (n=12 of 507). N= 1 ◊ MRSA	No other preventive measures were taken during the surgery and operation rooms were similar.<br><br>De study design did not include randomisation or blinding. Two cohorts are being compared.
Josefsson, 1993	A2	Randomised prospective controlled analysis control	Hip◊ 1688, Patients ◊ 1599	March 1976 - May 1978<br><br>487 patients died during study period. ◊<br>SA: 239 (29%)<br>GBC: 248 (29%)<br><br>Lost to follow-up:<br>Total: 86 hips<br>SA: 46<br>GBC:40	Female: 816<br>Mean age at surgery:<br>70 years (range 25-98 years)<br><br>Man: 783<br>Mean age surgery:<br>68 years (range 25-84 years)	SA (systemic antibiotics)<br>n= 835<br><br>Cloxacilline 1g 4x daily for 7-14 days, N= 359 hips<br><br>Cloxacilline 1g 4x daily for 8-14 days, N= 192 hips<br><br>Cephalexin 1g 4x daily for 9-11 days, N= 209 hips<br><br>Phenoxymethyl penicillin 0,65g 4x daily for 10 days, N= 75 hips	GBC (gentamine bone cement)<br>N= 835	10 years<br>N= 115 hips<br>SA<br>N= 550<br><br>GBC<br>N= 565	Deep wound infections	1 and 2 year study<br>16 deep wound infections<br>SA/GBA<br>13 (1,6%)/<br>9 (0,4%)<br>*P*< 0,05<br><br>5 years study<br>23 deep wound infections<br>SA/GBA<br>16 (1,9%) /<br>7 (0,8%)<br>*P*< 0,05<br><br>10 years study<br>22 deep wound infections<br>SA/GBA<br>13 (1,6%) /<br>9 (1,1%)<br>NS	Randomised, not blinded

Reference	Level of evidence	Study type	number of patients	Inclusion criteria	Patient characteristics	Intervention	Control	Follow-up	Outcome measures	Results	Remarks
**Espehoug 1997**	B	cohort	10.905 primary THR	**Sept. 1987-1995; reported in Norwegian hip-register<br>-only patients who had surgery for primary hip-osteoarthritis<br>-no earlier surgery<br>-cement used in both components-only THR with Charnley (DePuy, Leeds, UK), Exeter (Howmedica International, Herouville, France), Titan (Landos, Chaumont, France) or Spectron/ITH (Spectron acetabulum, ITH femur; Richards Memphis, Tennessee) component Cement ◊ high-viscosity Palacos or Simplex<br>-only common types of systemic antibiotics: cephalothin (n=6168), cefuroxime (n = 1969), dicloxacillin (n = 1468) and cloxacillin (n = 785)<br>-Antibiotics containing cement: gentamicine in combinative with Palacos (0,5 g per**	**1) <u>combined</u><br>n= 5804<br>male: 31%<br><65 years: 15%<br>65-74 years: 49%<br><br>2) <u>systemic</u><br>n= 4586<br>male: 30%<br><65 years: 17%<br>65-74 years: 52%<br><br>3) <u>bone cement</u><br>n= 239<br>male: 31%<br><65 years: 15%<br>65-74 years: 49%<br><br>4) <u>no antibiotics</u><br>n= 276<br>male: 30%<br><65 years: 17%<br>65-74 years: 53%**	**1) patients receive both systemic as local antibiotics prophylaxis in the bone cement<br>2) patients who received systemic antibiotics prophylaxis alone<br>3) patients who received antibiotics prophylaxis in the bone cement alone<br>4) no antibiotics prophylaxis**	**n.a.**	**7 years**	**Survival<br><br>Revision likelihood**	**Survival is shown in a figure, percentages are difficult to extract. The combined systemic + cement antibiotics result in best survival, followed by systemic alone.<br><u>Infection as end stage (5 jr. failure prob. %; 95% CI)/ number of revisions</u><br>1) 0,2 (0,1-0,4)/ 8<br>2) 0,8 (0,5-1,1)/ 25<br>3) 0,9 (0,0-2,0)/ 3<br>4) 1,2 (0,0-2,5)/ 3<br><br><u>Aseptic loosening as end stage (5 jr. failure prob. %; 95% CI)/ number of revisions</u><br>1) 1,0 (0,7-1,4)/ 44<br>2) 1,9 (1,3-2,4)/ 54<br>3) 2,1 (0,0-4,1)/ 7<br>4) 1,7 (0,0-3,4)/ 4<br><br><u>Any end stage (5 jr. failure prob. %; 95% CI)/ number of revisions</u><br>1) 1,6 (1,2-2,0)/ 70<br>2) 3,1 (2,4-3,8)/ 94<br>3) 2,9 (0,5-5,2)/ 10<br>4) 2,9 (0,7-4,9)/ 7**	

Reference	Level of evidence	Study type	number of patients	Inclusion criteria	Patient characteristics	Intervention	Control	Follow-up	Outcome measures	Results	Remarks
				**40,0 g polymethylmethacrylate; n = 5898) and erythromycin/colistine with Simplex cement (0,5 g erythromycin en 0,24 g colistine per 40,0 g polymethyl-methacrylate; n = 145) -infection as primary reason for revision**							

What is the preferred method to prevent postoperative thromboembolic complications?

Reference	Level of evidence	Study type	Number of patients	Patient characteristics	Intervention	Control	Follow-up duration	Outcome measures	Results	Remarks
Eriksson 2008	A2	RCT	3153	total hip arthroplasty	Rivaroxaban	Enoxaparine	36 days	Asymptomatic DVT, non-fatal pulmonary embolism and death	1,1% vs 3,7% ARR: 2.6%; 95% BI 1.5 - 3.7	Rivaroxaban is more effective and is equally safe with a long treatment duration
Eriksson 2007	A2	RCT	3494	total hip arthroplasty	Dabigatran etexilate 220mg and 150mg	Enoxaparine	33 days		6.0% (220mg) 8,6% (150mg) 6,7 (enoxaparine)	All treatment strategies are equally effective with similar safety.

What is the value physical therapy?

Reference	Level of evidence	Study type	Number of patients In/exclusion	Patient characteristics	Intervention/ Control	Outcome measures	Results	Remarks
**Preoperative physiotherapy**								
**Gocen, *2004***	B	RCT	**Study group**<br>N= 29<br><br>Control group<br><br>N= 30	Study group<br>**_: 13**<br>**_: 16**<br>**mean age: 46,93 ± SD 11, 48 years**<br>**BMI: 24,94 ± SD 3,7 kg/m^2**<br><br>Control group<br>**_: 8**<br>**_: 22**<br>**mean age: 55,5 ± SD 14,44 years**<br>**BMI: 27,69 ± SD 3,7 kg/m^2**<br><br>**Significant difference between both groups for age (*p*= 0,01)**	Study group<br>**Eight weeks preoperative strength and mobility training for hip muscles and information on how to live with an implant.**<br><br>Control group<br>**no training- or information programm**<br><br>**Both groups received similar postoperative guidance**	**_ Harris Hip Score**<br>**_ VAS-pain score (visual Analogue scale)**<br>**_ hip abduction range**<br><br>**Variables were measured at baseline (=8w preop, only intervention Group), at dismissal from the hospital, 3 months and 2 years postoperative.**	Harris Hip Score<br>Study group<br>**8 weeks preoperative: 42,7 ± 16,9**<br>**preceding surgery: 51,48 ± 18,32**<br>**at hospital dismissal: 64,46 ± 6,92**<br>**3 months postoperative: 85,30 ± 11,78**<br>**2 years postoperative: 97,14 ± 4,32**<br><br>Control group<br>**8 weeks preoperative: -**<br>**preceding surgery: 45,30 ± 12,98**<br>**at hospital dismissal: 59,36 ± 6,82**<br>**3 months postoperative: 78,70 ± 9,41**<br>**2 years postoperative: 95,66 ± 6,08**<br><br>**Day (sd) resume activity:**<br>Study group<br>**Walking: 2,07±0,20**<br>**Climbing stairs: 6,17±1,69**<br>**Getting out of bed: 2,93±0,59**<br>**Going to toilet: 4,24±0,51**<br>**Rise from chair: 4,24±0,74**<br><br>Control group<br>**Walking: 2,20±0,41**<br>**Climbing stairs: 7,37±1,02**<br>**Getting out of bed: 3,33±0,71**<br>**Going to toilet: 5,07±1,28**<br>**Rise from chair: 5,60±1,45**<br><br>**Statistically significant**	**Patients randomly assigned with Excel random numbers. even numbers ◊ control group**<br>**Odd numbers ◊ study group .**<br><br>**The assessing physiotherapist was blinded. Blinding of patients and treating therapist are not mentioned. patients**<br>**In de study group was 1 'drop out. All other patients were analysed in the group they were randomized to.**<br><br>**Significant difference between both groups concerning age. (*p*= 0,01)**<br><br>**The study groups were rather small, it is not certain the results can be generalized.**<br><br>clinical message:<br>**Routine preopative physiotherapy and education programs are not useful for patients with who undergo total hip replacement surgery.**

Reference	Level of evidence	Study type	Number of patients In/exclusion	Patient characteristics	Intervention/ Control	Outcome measures	Results	Remarks
							**difference: climbing stairs, getting out of bed, going to toilet, rise from chair: (respectively: 0,01; 0,02; 0,02; 0,001)**	
**Rooks, 2006**	B	RCT	**49 patients**<br><br><u>Intervention group</u><br>**N= 25**<br><u>Control group</u><br>**N= 24**	<u>Intervention group</u><br>**_: 63%**<br>**_: 37%**<br>**mean age: 65 ± SD 11 years**<br>**BMI: 28,4 ± SD 5,3 kg/m^2**<br><br><u>Control group</u><br>**_: 52%**<br>**_: 48%**<br>**mean age: 59 ± SD 7 years**<br>**BMI: 30,3 ± SD 9,1 kg/m^2**	<u>Intervention group</u><br>_ **3x weekly water and floor exercise**<br>_ **period: 6 weeks preop - surgery**<br><br><u>Control group</u><br>**Written information on home adjustments (increase accessibility and decrease fall accidents) and preparing for surgery by 3 telephone conversations, 2x written information via mail. Period: 6 weeks-preop – surgery.**	<u>Primary outcome measure</u><br>_ WOMAC function<br><br><u>Secondary Outcome measures</u><br>_ WOMAC pain score<br>_ SF-36<br>_ Lower-extremity strength<br>_ Balance<br>_ Mobility<br><br>Evaluated at 4 points in time:<br>_ pre-intervention<br>_ post-intervention (directly preoperative)<br>_ 8 weeks postoperative<br>_ 26 weeks postoperative	**Mean ± SD**<br>*Baseline*<br><u>Intervention group</u><br>**WOMAC function**<br>**29,1 ± 12,9**<br><u>Control group</u><br>**WOMAC function**<br>**29,8 ± 11,2**<br><br>*Preoperative**<br><u>Intervention group</u><br>**WOMAC function**<br>**26,9 ± 11,9**<br><u>Control group</u><br>**WOMAC function**<br>**33,7 ± 101,9**<br><br>*8 weeks postoperative*<br><u>Intervention group</u><br>**WOMAC function**<br>**12,8 ± 9,0**<br><u>Control group</u><br>**WOMAC function**<br>**12,9 ± 8,0**<br><br>*26 weeks post operative*<br><u>Intervention group</u><br>**WOMAC function**<br>**5,4 ± 5,8**<br><u>Control group</u><br>**WOMAC function**<br>**5,3 ± 5,4**<br><br>***statistically significant difference between the groups (*p*< 0,05)**	**Loss to follow-up > 20%.**<br><br>Randomisation and blinding procedures Procedures are not described, it is stated that it is a randomised blinded controlled study.

Reference	Level of evidence	Study type	Number of patients In/exclusion	Patient characteristics	Intervention/ Control	Outcome measures	Results	Remarks
**Ferrara, 2008**	B	RCT	**23 patients**<br><br>Exclusion criteria:<br>**Cognitive degeneration assessed with mini-mental State Examination ≤ 23, de presence of other joint implants, hip dysplasia, inflammatory arthritis , Parkinson and neuropathy.**	**End stage arthritis, patients were on a waiting list for total hip replacement**<br><br>Intervention group<br>**_: 7**<br>**_: 4**<br>**mean age: 63,82 ± SD 9,01 years**<br><br>Control group<br>**_: 7**<br>**_: 5**<br>**mean age: 63,08 ± SD 6,89 years**	Intervention group<br>**_ 1 month preop group- (40 minutes) and individual (20 minutes) exercises**<br>**_ 60 minutes daily**<br>**_ 5 days per week**<br>**_ Also exercises for muscle strength and flexibility**<br>**_ Received information on how to deal and exercise with a hip implant.**<br>**_ 4 weeks postop recovery program with exercise in hospital**<br><br>Control group<br>**Only 4w postop recovery program in hospital**	**_ Barthel Index**<br>**_ Short Form-36 (SF-36)**<br>**_ WOMAC**<br>**_ Harris Hip Score (HHS)**<br>**_ Muscle strength**<br>**_ Flexibility**<br>**_ VAS-pain score**<br><br>**Measurements were taken in all patients :**<br>**_ 1 months preop (T0)**<br>**_ 1 day preop (T1)**<br>**_ 15 days postop (T2)**<br>**_ 4 weeks postop (T3)**<br>**_ 3 months postop (T4)**	**The study group and de control group showed statistically significant results for VAS (*p*= 0,04), ROM external rotation (*p*= 0,03), SF-36 physical composite score (*p*= 0,048) and hip abductor (*p*= 0,004) at T1**<br><br>**On all other time points there were no significant differences for all outcome measures except for VAS and ROM external rotation at T4.**<br><br>**Mean ± SD**<br>Study group **T4**<br>**VAS***<br>**0,30 ± 0,48**<br>**ROM External rotation***<br>**33,50 ± 4,11**<br><br>Control group **T4**<br>**VAS***<br>**1,27 ± 1,00**<br>**ROM External rotation***<br>**33,64 ± 4,52**<br><br>****p*= < 0,05**	**The patients were randomised by using a randomising-table.**<br><br>**De assessors of effect were blinded. The physiotherapist who guided the exercise program was not blinded. Patient blinding is unknown.**<br><br>**Small study population, it is not certain results can be generalized.**
**Post operative physiotherapy**								
**Suetta, 2004**	B	RCT	**168 patients on the waiting list, 86 matched the inclusion criteria, 36 consented to participate in the study 30 patients completed follow-up.**<br>**SR - 3 patients**<br>**SRW -1 patient**<br>**NES - 2 patients**<br>**(see column intervention/Control for meaning)**<br><br>Inclusion<br>**Patients on the waiting list for a primary unilateral total hip**	SR (n=12)<br>**Mean age (years):**<br>**68 ± SD 62-78**<br>**_/_: 7/5**<br>**Mean weight (kg):**<br>**81,3 ± SD 5,8**<br>**Mean height (cm):**<br>**169,8 SD 2,1**<br>**BMI (kg/m^2):**<br>**28,2 ± SD 1,7**<br><br>SRW (n=13)<br>**Mean age (years):**<br>**69 ± SD 60-86**<br>**_/_: 6/7**	Intervention group<br>**3 groups:**<br>**_Standard rehabilitation at home (SR)**<br>**_Standard rehabilitation plus resistance-training (SRW)**<br>**_Standard rehabilitation plus Neuromuscular Electrical Stimulation (NES)**<br><br>**De SRW group and the NES group followed extra training or ES with the affected leg, in this way the non-affected leg could be the within-subject**	**Hospital stay**<br>**Muscle function, Muscle mass, Muscle strength**	Hospital stay<br>**SRW :** **10 ± 2,4 days, range 8-14)**<br>**SR:** **16 ± 7,2 days, range 9-35**<br>**SRW and SR differed significantly (*p*< 0,05)**<br>**NES:** **12 ± 2,8 days, range 8-16**<br><br>Muscle function (after 12 w)<br>*all values compared to baseline*<br>**SR**	**Randomisations with the aid of a computer program, patients were stratified according to age and gender.**<br><br>**Treating caregiver is blinded, blinding of patients and assessors is unknown. Comparable groups at baseline, small study population. ◊ generalisability is questionable**<br><br>**ITT analysis (though there [illegible]**

Reference	Level of evidence	Study type	Number of patients In/exclusion	Patient characteristics	Intervention/ Control	Outcome measures	Results	Remarks
			primary unilateral total hip replacement.<br><br>Age: 60 years or older, ASA score I or II<br>Excluded:: patients with cardiopulmonary, neurological or cognitive problems.	Mean weight (kg):<br>77,8 ± SD 4,5<br>Mean height (cm):<br>168,0 SD 2,0<br>BMI (kg/m^2):<br>27,4 ± SD 1,4<br><br>NES (n=11)<br>Mean age (years):<br>69 ± SD 60-75<br>_/_: 6/5<br>Mean weight (kg):<br>79,0 ± SD 4,2<br>Mean height (cm):<br>167,7 SD 2,8<br>BMI (kg/m^2):<br>27,9 ± SD 0,9	could be the within-subject control.<br><br>Control group<br>n.a.		No improvement<br>SRW<br>Improved: walking speed 30% ($p< 0,001$)<br>Climbing stairs 28% ($p< 0,005$)<br>Sit-to stand test 30% ($p< 0,001$)<br>NES<br>Improved: walking speed 19% ($p< 0,05$)<br>Climbing stairs 21% ($p< 0,001$)<br>Sit-to stand test 21% ($p< 0,001$)<br><br>Muscle mass<br>SR<br>Decline:<br>13% after 5 weeks ($p<0,05$)<br>9% after 12 weeks ($p<0,05$)<br>SRW<br>Stable after 5 weeks<br>Improved<br>12% ($p< 0,05$) after 12 weeks<br>NES<br>Decline 4% in 5 weeks ($p< 0,05$)<br>Improvement of 7% in 12 weeks ($p< 0,05$)<br><br>Muscle strength<br>SR<br>no change<br>SRW<br>Improved 22-28% ($p<0,05$ compared to baseline) after 12 weeks<br>NES<br>no change	were dropouts)
Trudelle, 2004	B	RCT	Study group<br>N= 14<br><br>Control group	Study group<br>mean age (years):<br>59,4 ± SD 10,8<br>Mean weight (kg):<br>83,0 ± SD 17,2	Intervention group<br>strength- and stability exercises<br><br>Control group<br>isometric strength and active	– 12 Item Hip<br>– Questionnaire<br>– fear of fall<br>– hip flexors,<br>– hip extensors	% change in muscle strength and stability after 8 weeks<br><br>Study group<br>Stability 36,8*<br>Hip flexors 24,4*	The study design is single blind randomised study. Patients were randomized with a random number table. Patients were blinded, therapist was also blinded

Reference	Level of evidence	Study type	Number of patients In/exclusion	Patient characteristics	Intervention/ Control	Outcome measures	Results	Remarks
			**N= 14**<br><br>**<u>Inclusion</u>**<br>4 to 12 months post operative total hip replacement	**Mean height (cm): 169,1 ± SD 7,6**<br><br><u>Control group</u><br>**mean age (years): 59,6 ± SD 12,1**<br>**Mean weight (kg): 80,4 ± SD 18,9 Mean height (cm): 170,5 ± SD 10,2**	**mobility training**	**– hip abductors**<br>**– knee-extensors**<br>**– stability (stand on 1 leg)**	**Hip extensors 47,8***<br>**Hip abductors 41,2***<br>**knee-extensor 23,4***<br><br><u>Control group</u><br>**Stability 0,9**<br>**Hip flexors 7,2**<br>**Hip extensors 3,6**<br>**Hip abductors 3,3**<br>**knee-extensors 1,0**<br><br>*** p≤0,05 (difference between pre- and post exercise)**	**therapist was also blinded. Blinding of the assessor is not described.**<br><br>**The number of patients included in the study is small.**
**Maire, 2006**	C	RCT (Pilot study)	**14 patients**<br><br><u>Inclusion</u><br>**Volunteers 65 years or older with primary hip osteoarthritis as main diagnose.**	<u>Study</u><br>**N= 14**<br>**mean age (years): 75,1 ± SD 4,8**<br>**Mean weight (kg): 73,8 ± SD 13,5**<br>**Mean height (cm): 158,2 ± SD 7,9**<br>**BMI (kg/m^2): 29,3 ± SD 4,7**<br><br><u>Intervention group</u><br>**N= 7**<br>**_/_: 1/6**<br><u>Control group</u><br>**N= 7**<br>**_/_: 1/6**	<u>Intervention group</u><br>**Besides the normal rehabilitation program, an interval training program of the arms (3 sessions a week)**<br><br><u>Control group</u><br>**Normal rehabilitation program alone.**	**Primary outcome measures**<br>**– walking distance in 6 minutes, measured after 2 and after 12 months postoperative**<br>**– WOMAC total and Physical Function (before surgery and after 2 and 12 months)**	**<u>walking distance after two months</u>**<br>Intervention group :<br>**mean 396 meters**<br>Control group :<br>**mean 268 meters**<br>**$p < 0,05$)**<br>**This difference was declined after one year, but still significant (mean value: 494 vs 406 m, $p < 0,05$).**<br><br>**Both groups improved on all WOMAC-aspects after 2 months and after one year in comparison with the preoperative outcomes ($p <$ 0,05).**<br>**However, the intervention group had lower WOMAC-scores than the control group ($p < 0,05$).**	**Small study population, compromises generalisability.**<br><br>**No procedures for randomisation and blinding of outcome assessor are described.**
**Galea, 2008**	B	RCT	**23 patients**<br><br><u>Inclusion</u><br>**Uncomplicated unilateral THR; primary diagnosis: hip OA**<br>**- being able to walk 45m independently**<br>**-independently Sit-to-stand transfer**<br>**-able to adequately understand written and oral instructions.**	***I= intervention***<br>***C=Control***<br>***Mean ± SD***<br>Gender (_/_)<br>**I:3/8**<br>**C:4/8 NA**<br>Age (years)<br>**I: 68,6±9,7**<br>**C:66,6±7,9 *p=0*,55**<br>Weight (kg)<br>**I: 76,3±14,4**<br>**C:81,6±20,3 *p=0*,47**	<u>Intervention group</u><br>**'supervised training group (in hospital or rehabilitation centre)**<br>**(n= 12)**<br><br>**Twice weekly; 45 min. Received extra instructions about exercise progression from a physiotherapist in 2 sessions.**	**TUG test**<br>**Climbing stairs**<br>**6MWT**<br>**physical function**<br>**Quality of Life**	**Intervention group practiced 4,7x compared to 5,8 x for the control group. NS after 8 weeks◊ physical function, climbing stairs, 6MWT test and quality of life (F = 0,438, *p*= 0,9)**<br><br>**TUG test ◊ sig. difference between both group en after 8 weeks (*p*= 0,042)**	**No real control group (Placebo)**<br><br>**Randomised, unknown how.**<br><br>**No information on blinding of outcome assessor.**<br><br>**Small study population**

Reference	Level of evidence	Study type	Number of patients In/exclusion	Patient characteristics	Intervention/ Control	Outcome measures	Results	Remarks
				Height (m)<br>**I: 1,6±0,1**<br>**C:1,6±0,1 *p=0,*83**<br>BMI (kg/m2)<br>**I: 28,1±4,5**<br>**C: 29,6±5,2 *p=0,*49**<br>Affected side (left/right)<br>**I: 5/6**<br>**C: 6/6 NA**	**7 exercises: (Figure-or-eight path walk, Sit to stand, Active single-leg stance, Climbing steps, Hip abduction, Heel raise, Side stepping.)**<br><br>Control group<br>**At home training**<br>**(n= 11)**<br><br>**Written guidance, similar exercises as intervention group.**			
**Unlu, 2007**	B	RCT	**26 patients**<br><br>Inclusion<br>**12 -24 months postoperative**<br><br>Exclusion:<br>Neurological, cognitive or metabolic diseases, early postoperative complications, revisions or other joint problems which may cause difficulties in moving the patient.	Group 1 **(n= 9)**<br>**Mean age (years):**<br>**45,44 ± SD 8,7**<br>**_/_: 7/2**<br>**Mean weight (kg):**<br>**77,55 ± SD 4,74**<br>**Mean height (cm):**<br>**162,11 ± SD 6,75**<br>**Mean age implant (months):**<br>**17 ± SD 6,16**<br>**Implant side (L/R): 5/4**<br><br>Group 2 **(n= 8)**<br>**Mean age (years):**<br>**57,75 ± SD 7,45**<br>**_/_: 6/2**<br>**Mean weight (kg):**<br>**73,25 ± SD 9,11**<br>**Mean height (cm):**<br>**158,75 ± SD 7,70**<br>**Mean age implant (months):**<br>**19 ± SD 8,05**<br>**Implant side (L/R): 4/4**<br><br>Group 3 **(n= 9)**<br>**Mean age (years):**<br>**52,55 ± SD 10,32**<br>**_/_: 5/4**<br>**Mean weight (kg):**	Group 1<br>**Exercise program at home ◊ mobility and muscle strength exercises**<br>**Twice daily, 6 weeks consecutively.**<br>**Experienced physiotherapist explains exercises in practice lesson. 1 weekly contact with physiotherapist.**<br><br>Group 2<br>**Similar exercise programmes group 1, but executed under the supervision of a physiotherapist in the hospital.**<br><br>Group 3<br>**Walking**	Primary outcome:<br>**strength**<br><br>**Other outcome measures:**<br>**Walking speed (meters per minute), cadence (number of steps per minute)**	*All p-values compared to baseline*<br>**after 6 weeks**<br>Group 1 **(mean ± SD)**<br>Strength (ft. lb)<br>**Begin: 30 ± 12**<br>**End: 38 ± 11**<br>***p-values*: 0,018**<br>Walking speed (m/min)<br>**Begin: 67,8 ± 23**<br>**End: 74,35 ±24**<br>***p-values*: 0,021**<br>Cadence (steps / minute)<br>**Begin: 97,7 ± 18**<br>**End: 111 ± 17**<br>***p-values*: 0,011**<br><br>Group 2 **(mean ± SD)**<br>Strength (ft. lb)<br>**Begin: 18 ± 10**<br>**End: 30 ± 9,8**<br>***p-values*: 0,012**<br>Walking speed (m/min)<br>**Begin: 48,53 ± 4**<br>**End: 56,7 ± 5**<br>***p-values*: 0,012**<br>Cadence (steps/ minute)<br>**Begin: 90,75 ± 6**<br>**End: 104 ± 7**<br>***p-values*: 0,012**<br><br>Group 3 **(mean ± SD)**<br>strength (ft. lb)<br>**Begin: 18 ± 10**<br>**End: 19 ± 8**	**80 patients were recruited of which 22 were excluded..Of the remaining 58 patients, 32 could not be randomised (12 unable to reach by phone), 20 dropped out because of financial issues..**<br>**The remaining 26 patients were randomised (32,5 % of total)**<br>**Low number for generalisability.**<br><br>**Other limitations: short rehabilitation period (6 weeks)**<br><br>**Strengths of the study:**<br>**Randomisation**<br>**Blinding of outcome assessor, patients and therapist.**<br>**(Closed envelopes with a symbol corresponding with a treatment group, using a list of numbers which were randomly generated.**<br><br>**ITT analysis**<br><br>**There was a significant difference for mean age between Group 3 and the other groups.**

Reference	Level of evidence	Study type	Number of patients In/exclusion	Patient characteristics	Intervention/ Control	Outcome measures	Results	Remarks
				**72,44 ± SD 12,61**<br>**Mean height (cm):**<br>**162,78 ± SD 9,17**<br>**Mean age implant (months):**<br>**16,55 ± SD 8,51**<br>**Implant side (L/R): 4/5**<br><br>**Significant difference forage** ***p*=0,033**			***p-values*: 0,200**<br>Walking speed (m/min)<br>**Begin: 58,01 ± 12**<br>**End: 59,8 ± 14**<br>***p-values*: 0,110**<br>Cadence (steps/ minute)<br>**Begin: 87 ± 16**<br>**End: 88,22 ± 16**<br>***p-values*: 0,119**<br><br>**There were not significant differences between group 1 and 2. Walking speed and cadence were significantly different in group 3 compared to group 1 and 2.**	**other groups.**
**Jan, 2004**	B	RCT	**53 patients randomised**<br><br>Intervention group<br>**n=29**<br>**lost to follow-up n=3**<br>**analyze n=26**<br><br>Control group<br>**n=29**<br>**lost to follow-up n=2**<br>**analyze n=27**<br><br>Inclusion<br>**Undergoing a primary total hip replacement 1,5 years or more before start of the study, executed by the same surgeon who used the anterolateral technique, no revision afterwards and able to walk without support.**	Intervention group (low compliance) **(n=13)**<br>**Mean age (years):**<br>**59,3 ± SD 10,3**<br>**_/_: 8/5**<br>**Mean weight (kg):**<br>**142,4 ± SD 22,7**<br>**Mean height (cm):**<br>**158,5 ± SD 4,6**<br>**Months after THR:**<br>**72,2 ± 51,6**<br>**Affected side (L/R): 7/5**<br><br>Intervention group (high compliance)**(n=13)**<br>**Mean age (years):**<br>**58,8 ± SD 12,9**<br>**_/_: 9/4**<br>**Mean weight (kg):**<br>**137,7 ± SD 22,2**<br>**Mean height (cm):**<br>**159,5 ± SD 7,6**<br>**Months after THR:**<br>**54,2 ± 46,5**<br>**Affected side (L/R): 7/6**<br><br>Control group	Intervention group<br>**-At home exercise program; hip flexibility exercises, strength exercises, 30 minutes walking training on average/low speed.**<br><br>Control group<br>**-No intervention**	- **Muscle strength (measured with dynamometer)**<br>- **Walking speed (vrij and vast) op 3 different terrains (video-opnames)**<br>- **functioning (Harris Hip Score)**	**De intervention group (high compliance) improved on all outcome measures significantly (*p*= 0,05) better than the control group and the low compliance group after 12 weeks.**	**Patients were randomly assigned to the intervention group or control Group. Blinding of the outcome assessor is not mentioned.**<br><br>**50% of the intervention group showed low compliance.**<br><br>**Difference between the intervention group (high and low compliance) and de control group was significant for height (*p*= 0,05)**

Reference	Level of evidence	Study type	Number of patients In/exclusion	Patient characteristics	Intervention/ Control	Outcome measures	Results	Remarks
				**Mean age (years):**<br>**57,0 ± SD 18,8**<br>**_/_: 10/17**<br>**Mean weight (kg):**<br>**141,8 ± SD 21,4**<br>**Mean height (cm):**<br>**163,0 ± SD 9,7**<br>**Months after THR:**<br>**76,0 ± 52,0**<br>**Affected side (L/R):**<br>**14/13**<br><br>**Difference between the groups is significant for height (*p*= 0,05)**				

How to prevent haematogenous infection of prosthesis?

Reference	Level of evidence	Study type	Number of patients In/exclusion	Patient characteristics	Intervention/ Control	Outcome measures	Results	Remarks
**Ainscow, 1984**	C	Case series	1000 patients<br>1966-1980	**284 male**<br>**716 female**<br>**1112 total joint prostheses**<br>**Mean age was 70 years (range 49-85 years)**<br>**Primary or secondary osteoarthritis (n=866)**<br>**Rheumatoid or the like (n=134)**	**No instructions for antibiotics tooth surgeries or other surgeries or in case of infections (antibiotics only administered on basis of clinical indication)**<br><br>***Follow-up***<br>**mean 6 years (range; 3-15 years)**	**Deep infections**<br><br>**Hematogenic infection**	**22 joints developed deep infection: 11 within 3 months, 8 after 3 months (non-hematogenic)**<br><br>**3 were caused by hematogenic infection Overall incidence was 0,27% in 6 years. Annual incidence was 0,04%**<br><br>**Of the 134 rheumatoid arthritis patients, 2 developed hematogenic infection (1,5%; *p* < 0,05)**<br><br>**450 patients had no risk**<br>**224 patients had tooth**	

Reference	Level of evidence	Study type	Number of patients In/exclusion	Patient characteristics	Intervention/ Control	Outcome measures	Results	Remarks
							**surgery or other surgery ◊ none of these patients developed a hematogenic infection<br>288 patients developed a urine tract, lung- or other infection ◊ some had tooth surgery, none of them developed a hematogenic infection.<br>Of the 40 patients whose skin was ulcerated and infected, 3 pt developed a hematogenic infection. (7,5%; *p*< 0,01)**	
**Krijnen, 2001**	B	Cost- effectiveness study	4907 patients<br>Joint disease<br>Amsterdam<br>Data from prospective study on bacterial arthritis were combined with data from literature to examine risks and advantages.<br><br>**Effectiveness and cost-effectiveness of antibiotics was assessed in different groups/patients.<br>Groups based on (*a*) type of infection (skin-, lung- or urine tract infection) and invasive medical procedure and (*b*) the patient sensitivity for bacterial arthritis, which was present in the form of rheumatoid arthritis, larger joint prostheses, comorbidity, and older age.**	**14 of the 37 were hematogenic**	**Administering antibiotics (n= 37)/ no antibiotics (n=4870)**	**Cost-effectiveness of antibiotics prophylaxis for hematogenic bacterial arthritis**	**59% had no characteristics for sensitivity for bacterial arthritis, and 31% had 1.<br><u>Skin infection</u><br>Effectiveness off antibiotics was maximal 35 quality Adjusted life days (QALDs) and de cost-effectiveness max. $52 000 per quality adjusted life year (QALY).<br><u>Other infections</u><br>Effectiveness of antibiotics was lower and de cost-effectiveness was higher.<br>Antibiotics prophylaxis for invasive medical use seems acceptable for patients with high sensitivity:<br>1 QALD at costs of $1300/QALY<br><br>De results affected sensitivity when the effect of the prophylaxis or the cost of the prophylaxis changed.**	**Knee and hip prosthesis patients, no separate data**
**Kaandorp, 1998**	?	Is a thesis						
**Uckay, 2008**	**D**	Is no original study or meta-analysis with data collection and analysis, but current						

Reference	Level of evidence	Study type	Number of patients In/exclusion	Patient characteristics	Intervention/ Control	Outcome measures	Results	Remarks
		concepts review.						
**Decon, 1996**	**D**	Is no original study or meta-analysis with data collection and analysis, but current concepts review.						
**Rompen, 2008**	**D**	Is no original study or meta-analysis with data collection and analysis, but current concepts review.						
**Abraham-Inpijn, 2005**	**D**	Is no original study or meta-analysis with data collection and analysis, but current concepts review.				–		
